# Electron Microscopy and Multi‐Omics Reveal Mitochondrial Dysfunction and Structural Remodeling in the Hearts of Elderly Mice

**DOI:** 10.1111/acel.70286

**Published:** 2025-11-18

**Authors:** Manuela Giovanna Basilicata, Marco Malavolta, Serena Marcozzi, Eduardo Sommella, Lucia Scisciola, Fabrizio Merciai, Gianluca Fulgenzi, Valentina Golino, Giovanni Tortorella, Tatiana Spadoni, Laura Graciotti, Tania Ciaglia, Leonardo Schirone, Valentina Valenti, Sebastiano Sciarretta, Ceereena Ubaida‐Mohien, Carmine Pizzi, Rafael De Cabo, Pietro Campiglia, Lucia Altucci, Michelangela Barbieri, Fabiola Olivieri, Luigi Ferrucci, Giuseppe Paolisso

**Affiliations:** ^1^ Department of Advanced Medical and Surgical Sciences University of Campania “Luigi Vanvitelli” Naples Italy; ^2^ Advanced Technology Center for Aging Research and Geriatric Mouse Clinic, IRCCS INRCA Ancona Italy; ^3^ Department of Clinical and Molecular Sciences (DISCLIMO) Polytechnic University of Marche Ancona Italy; ^4^ Scientific Direction, IRCCS INRCA Ancona Italy; ^5^ Department of Pharmacy University of Salerno Fisciano Italy; ^6^ National PhD Program in RNA Therapeutics and Gene Therapy University of Naples Federico II Napoli Italy; ^7^ Department of Biomedical Sciences and Public Health Polytechnic University of Marche Ancona Italy; ^8^ Department of Health and Life Science European University of Rome Rome Italy; ^9^ Department of Medico‐Surgical Sciences and Biotechnologies University of Sapienza Rome Italy; ^10^ IRCCS Neuromed Pozzilli Italy; ^11^ Intramural Research Program, National Institute on Aging National Institutes of Health Baltimore Maryland USA; ^12^ Department of Medical and Surgical Sciences‐DIMEC‐Alma Mater Studiorum University of Bologna Bologna Italy; ^13^ Cardiovascular Division Morgagni–Pierantoni University Hospital Forlì Italy; ^14^ National Institute on Aging Baltimore Maryland USA; ^15^ Department of Precision Medicine University of Campania “Luigi Vanvitelli” Naples Italy; ^16^ Program of Medical Epigenetics Vanvitelli Hospital Naples Italy; ^17^ Biogem, Molecular Biology and Genetics Research Institute Italy; ^18^ UniCamillus International University of Health Sciences Rome Italy

**Keywords:** aging, cardiac dysfunction, heart, mitochondria, multi‐omics approach

## Abstract

Aging is a key driver of cardiac dysfunction, promoting structural remodeling, metabolic alterations, and loss of cellular resilience. In aged hearts, extracellular matrix remodeling and collagen accumulation reduce ventricular compliance, impairing both diastolic function and stress adaptability. Cardiomyocytes exhibit diminished regenerative capacity and dysregulated stress responses, with mitochondrial dysfunction emerging as a central contributor to energy imbalance, oxidative stress, and fibrosis. Traditional single‐omics approaches are insufficient to capture the complexity of these interconnected changes. To address this, we employed an integrative multi‐omics strategy—combining spatial transcriptomics, proteomics, and metabo‐lipidomics with electron microscopy—to investigate cardiac aging in mice at three life stages: adult (12 months), middle‐aged (24 months), and elderly (30 months). Electron microscopy revealed enlarged, structurally compromised mitochondria. Spatial transcriptomics showed reduced expression of cardioprotective genes (MANF, CISH, and BNP) and increased expression of profibrotic markers like CTGF. Proteomics revealed widespread mitochondrial dysregulation and impaired ATP production. Metabolic and lipidomic profiling identified reduced antioxidant metabolites and accumulation of lipotoxic species, such as ceramides and diacylglycerols. This multiscale analysis highlights key molecular and metabolic alterations driving cardiac aging, identifying potential therapeutic targets to mitigate age‐related functional decline. Overall, our findings highlight the value of integrated, system‐level approaches for uncovering the complex mechanisms that drive organ aging. Although our study was conducted in mice, validation in human models will be crucial to establish the translational relevance of these results and to guide future research with potential impact across diverse biomedical fields.

## Graphical abstract

Integrated multi‐omics mapping of cardiac aging reveals mitochondrial dysfunction, profibrotic reprogramming, and lipotoxicity as key drivers of myocardial decline. A comprehensive spatial and molecular analysis of mouse hearts across aging (12, 24, and 30 months) uncovers coordinated disruptions in gene expression, protein homeostasis, and lipid metabolism, pinpointing therapeutic targets to counteract age‐related cardiac dysfunction.

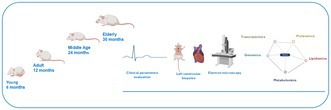

**Abbreviations**
ACTA1: Actin Alpha 1 Skeletal Muscle
ACTA2: Actin Alpha 2, Smooth Muscle
AGEs: Advanced glycation end‐products
ANKRD1: Ankyrin Repeat Domain 1
BGN: Biglycan Cellular Communication Network Factor 2
CERS: Ceramides
CFI: Clinical Frailty Index
CISH: Cytokine Inducible SH2 Containing Protein
COL3A1: Collagen Type III Alpha 1 Chain
CTGF: Connective tissue growth factor
DDI: Diastolic Dysfunction Index
DEG: Differentially expressed gene
DGs: Diacylglycerols
Dld: Dihydrolipoyl dehydrogenase
ECG: Electrocardiography
EM: Electron microscopy
GEE: Generalized estimating equations
GPx4: Glutathione peroxidase 4
GSH: Glutathione
IPA: Ingenuity pathway analysis
KEGG: Kyoto Encyclopedia of Genes and Genomes
LD: Lipid droplets
LFQ: Label‐free quantification
LGSH: Lactoyl glutathione
MANF: Mesencephalic Astrocyte‐Derived Neurotrophic Factor
mPTP: Mitochondrial permeability transition pore
Ndufa5, Ndufa7, Ndufv2: NADH dehydrogenases
NPPA: Natriuretic peptide A
NPPB: Natriuretic peptide B
OGDH: 2‐Oxoglutarate dehydrogenase
Pdha1: Pyruvate dehydrogenase E1
POSTN: Periostin
PPif: Peptidyl‐prolyl cis‐trans isomerase F
Prdx1: Peroxiredoxin 1
Proteasome components: Psmc2, Psmc3, Psmc4, Psmd2, Psmd4, Psmd13
RNS: Reactive nitrogen species
scRNA‐Seq: Single‐cell RNA sequencing
SMPDB: Small Molecule Pathway Database
Suclg2: Succinate–CoA ligase
TCA: tricarboxylic acid cycle
TIMM10: Translocase of Inner Mitochondrial Membrane 10
TNS1: Tensin 1
TUBA8: Tubulin Alpha 8
Usp15: Ubiquitin carboxyl‐terminal hydrolase 15
VWF: Von Willebrand Factor

## 1 Introduction

Extensive evidence from clinical and experimental studies suggests that the aging heart undergoes age‐related fibrotic remodeling (Reiman and Mintun 1990). Age‐dependent cardiac collagen accumulation progressively increases ventricular stiffness and impairs diastolic function (Biernacka and Frangogiannis 2011). This phenomenon is associated with increased ventricular wall thickness. Thus, the altered extracellular matrix composition contributes to impaired diastolic filling and reduced systolic function under stress (Cheng et al. 2009). Age‐related changes in cardiac myocytes include decreased cellular proliferation, age‐associated alterations in the cardiac microenvironment (such as changes in inflammatory signaling and oxidative stress), shifts in isomyosin expression from α‐ to β‐myosin heavy chain, increased expression of fibrosis‐related genes, and changes in growth‐controlling factors such as reduced endocrine factor and nerve growth factor response, insulin response, and increased expression of Angiotensin II receptor. Interestingly, mitochondrial dysfunction plays a central role in cardiac aging (Tocchi et al. 2015), as it impairs cellular energy production and elevates oxidative stress, thereby promoting damage to macromolecules such as proteins and lipids. Because of its high energetic demand and high density of mitochondria, an aged heart is especially vulnerable to mitochondrial dysfunction via structural disruption, energetic fluctuations, and mitochondrial signaling (Xie et al. 2023). Given the cardiac reliance on mitochondrial function for energy production, these alterations significantly impact cardiac myocytes, contributing to the age‐associated decline in cardiac performance. Various omics technologies are needed to comprehensively understand the molecular basis of structural and functional dysregulation in the aging heart (Dai et al. 2008; Qu et al. 2023; Tham et al. 2018). Despite increasing evidence implicating mitochondrial dysfunction in cardiac aging, the specific molecular mechanisms linking structural alterations to transcriptional, proteomic, and metabolic remodeling remain poorly understood. To address this gap, our study integrates electron microscopy with a multi‐omics strategy—combining spatial transcriptomics, proteomics, and metabo‐lipidomics—to provide a comprehensive view of how interconnected biological processes drive age‐related cardiac decline.

## 2 Methods

### 2.1 Animals and Experimental Design

The investigation conforms to the Guide for the Care and Use of Laboratory Animals published by the US National Institutes of Health (NIH Publication No. 85‐23, revised 1985). The Supporting Information reports information about the animals used in the study, the experimental design, biochemical parameters, Clinical Frailty Index (CFI), electrocardiography (ECG), and echocardiography analyses.

### 2.2 ECG‐Derived Diastolic Dysfunction Index

Electrocardiographic parameters associated with diastolic function were extracted from conscious mice at 12, 24, and 30 months of age. PR interval and heart rate variability (HRV) were selected on the basis of their reported relevance to diastolic dysfunction and autonomic regulation. Each parameter was normalized using *Z*‐score transformation (PRz and HRVz). An ECG‐derived Diastolic Dysfunction Index (DDI) was then calculated as: DDI = PRz—HRVz. Higher values of the DDI reflect more pronounced diastolic dysfunction, on the basis of the directionality of each parameter's known association with impaired diastolic performance both in humans (Van Ommen et al. 2021), (Namdar et al. 2013) and mice (Nascimento‐Carvalho et al. 2024), (Slotabec et al. 2024).

### 2.3 Electron Microscopy Data

Hearts from avertin‐anesthetized mice were rapidly excised, and approximately 1 mm^3^ of the posterior ventricular wall was dissected, briefly immersed in a myorelaxant solution, and fixed for 1 h at room temperature. The tissue was then washed, dehydrated, and embedded in Epon‐Araldite resin. Ultrathin sections (50 nm) were obtained and analyzed using a Philips CM12 transmission electron microscope. Mitochondria were manually outlined in ImageJ to measure morphometric parameters such as area, perimeter, major and minor axes, circularity index, cristae score and mitochondrial number (Lam et al. 2021; Neikirk et al. 2023). Statistical analyses were performed using Generalized Estimating Equations (GEE) with a gamma distribution to account for intra‐animal correlation and data distribution characteristics. Area and other variables' measurements were collected in multiple replicates per mouse across three experimental groups: Adult (12 months), Middle age (24 months), and Elderly (30 months). GEE models were fitted using a gamma distribution with a log link function to accommodate the positive skew and heteroscedasticity of the data. The mouse identifier (Mouse_ID) was included as the subject variable to adjust for intra‐animal correlation. All analyses were conducted using SPSS (Version 26, IBM Corp). Detailed parameters are reported in the Supporting Information.

### 2.4 Spatial Transcriptomics

Spatial transcriptomics was performed using the BMKMANU S1000 RNA‐seq platform (Biomarker Technologies (BMK) GmbH, Münster, Germany) with the BMKMANU S1000 Gene Expression kit. The Supporting Information includes extended methods, detailed tissue preparation, sequencing, and data analysis.

### 2.5 Proteome Extraction and Analysis

Proteome extraction was performed on 3 mg of lyophilized cardiac ventricular tissue, homogenized using a HT Lysing Homogenizer (OHAUS Europe GmbH, Nanikon, Switzerland). Protein quantification was conducted via the BCA method. Samples preparation followed the iST kit protocol (PreOmics, Martinsried, Germany). Proteomic analysis was performed as reported elsewhere (Scisciola et al. 2023); detailed parameters are provided in the Supporting Information—extended methods.

### 2.6 Metabolome and Lipidome Extraction and Analysis

Metabolome and lipidome extraction were performed on 1 mg of lyophilized ventricular tissue using MeOH/H_2_O (1:1 v/v), followed by sonication, centrifugation, and MTBE extraction with deuterated standards. Supernatants were dried using a SpeedVac. Metabolomic and lipidomic analyses were carried out as reported previously (Carbone et al. 2022). Detailed method parameters are in the Supporting Information—extended methods.

### 2.7 Clinical Frailty Index

To support the clinical relevance of the aging model, we applied the validated 31‐item Clinical Frailty Index (CFI) to all animals. This index captures a range of deficits across multiple systems including integument, musculoskeletal function, vestibulocochlear/auditory performance, digestive and urogenital health, respiratory status, and general discomfort.

### 2.8 Data Analysis

All clinical, ECG, and Echocardiographic data were expressed as ± SD. ANOVA calculated age‐related differences. Each omic dataset was pre‐processed independently. Proteomics and metabolomics data were normalized by total ion sum, whereas lipidomics data were normalized using class‐specific internal standards. Missing values were replaced with one‐fifth of the dataset's minimum value, followed by log_10_ transformation and autoscaling (Caponigro et al. 2023). The univariate analysis was conducted using N‐way ANOVA (*p* < 0.05). Enrichment analyses were carried out using STRING (https://string‐db.org/), ShinyGO 0.82 (http://bioinformatics.sdstate.edu/go/), and LipidOne 2.0 (https://lipidone.eu/).

## 3 Results

### 3.1 Age‐Related Changes in Biochemical and Clinical Markers of Health Correlated With Age Groups

**FIGURE 1:**
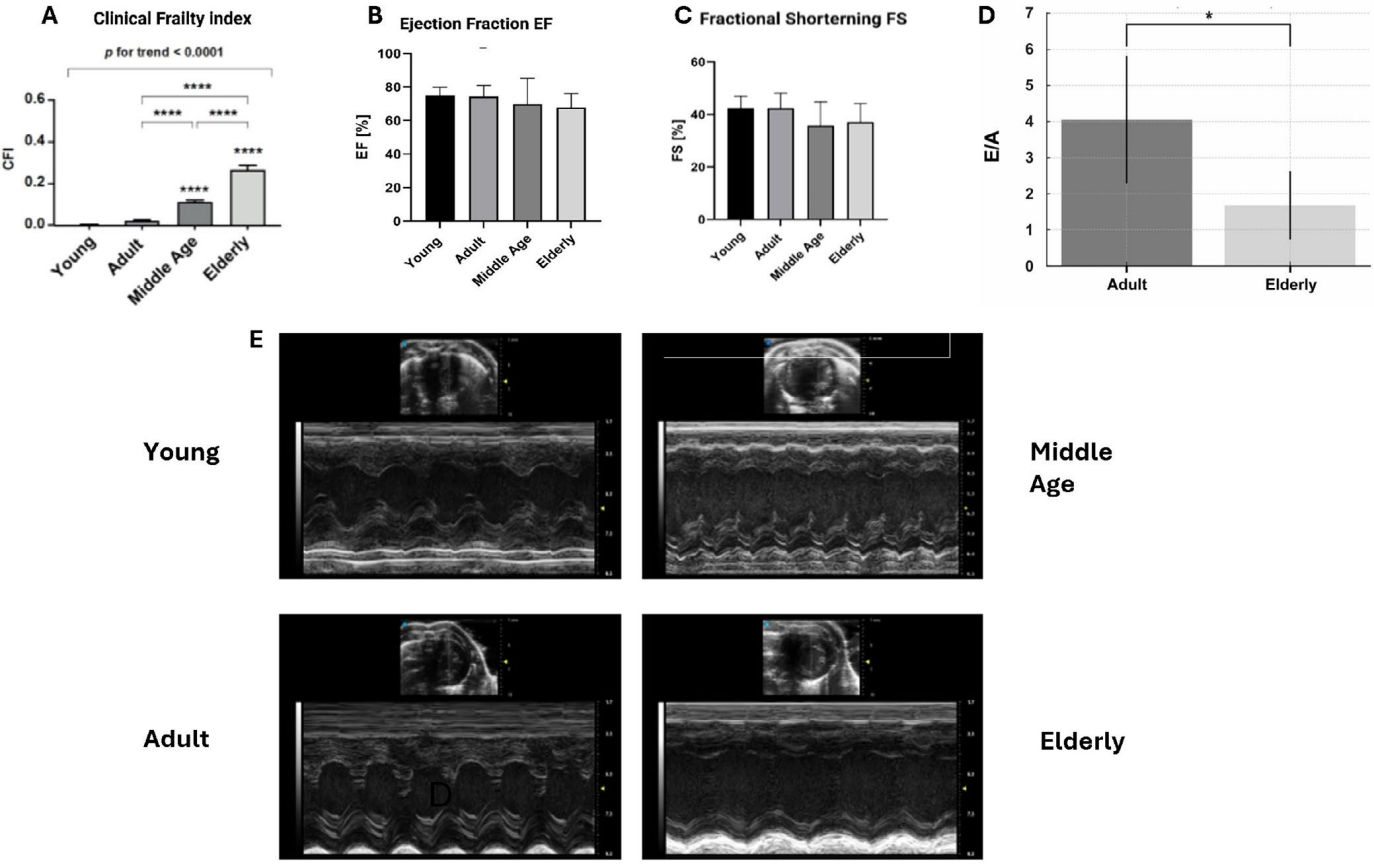
(A–E) (A) The Clinical Frailty Index (CFI) shows a progressive and statistically significant increase with age, with a highly significant trend (*p* for trend < 0.0001). Multiple comparisons indicate significant differences between all groups (Young, Adult, Middle Age, Elderly), ****p < 0.0001. The Young group is shown for reference, to illustrate that values between Young and Adult mice are comparable and that no significant differences are present at this early stage. This highlights that the major changes emerge only at later ages, whereas the transition from Young to Adult remains stable. (B) Ejection Fraction (EF) was also unchanged among groups, confirming the absence of systolic dysfunction associated with aging. (C) Fractional Shortening (FS) did not differ across age groups, indicating preserved left ventricular systolic function. (D) E/A ratio Across multiple tests (Welch *t*‐test, Student *t*‐test, Mann–Whitney *U*, permutation test, log‐transformed Welch, and winsorized Welch), the obtained results showed lower E/A in 30 m mice. *p*‐values ranged from 0.009 to 0.020, reaching statistical significance in all but the Kolmogorov–Smirnov distributional test. Effect sizes were large (Hedges' *g* = −1.55; Cliff's delta = −0.78), confirming a substantial biological difference. (E) Representative M‐mode echocardiographic recordings for each age group are displayed in the center of the figure.

Biochemical assessments confirmed animal health overall, with blood glucose, creatinine, and albumin levels within expected ranges across age groups. The CFI showed that among all mouse groups assessed, only the 30‐month‐old mice exhibited a pronounced frailty phenotype, consistent with a geriatric stage of biological aging (Marcozzi et al. 2023) (Figure 1A). Electrocardiography revealed no abnormalities in cardiac signal transmission beyond the expected progressive age‐related decline in heart rate (*p* for trend < 0.001). Echocardiographic evaluations depicted a non‐statistically significant age‐trend decline of fractional shortening (Figure 1B,C, Table S1). The DDI significantly increased with age (one‐way ANOVA, *p* < 0.05), suggesting progressive worsening of diastolic function from 12 to 30 months of age. Notably, mice at 30 months exhibited the highest DDI scores, consistent with advanced autonomic and electrical remodeling. The index was able to differentiate between early aging (12 vs. 24 months) and more severe dysfunction in late aging (30 months), supporting the presence of progressive diastolic dysfunction with advancing age in mice (Figure S1). To validate the ECG findings, we compared the E/A ratio between 12‐ and 30‐month‐old mice (*n* = 6 per group). Multiple statistical tests showed consistently lower E/A values in the older group (*p* = 0.009–0.020; large effect sizes: Hedges' *g* = −1.55, Cliff's delta = −0.78), indicating marked age‐related impairment of diastolic relaxation and suggesting elevated left ventricular filling pressures and increased myocardial stiffness (Figure 1D). Given the lack of significant differences between young and adult mice, we selected adult, middle‐aged, and elderly mice for subsequent multi‐omics and ultrastructural analyses.

### 3.2 Electron Microscopy Data Uncover Age‐Related Mitochondrial Structural Alterations

**FIGURE 2:**
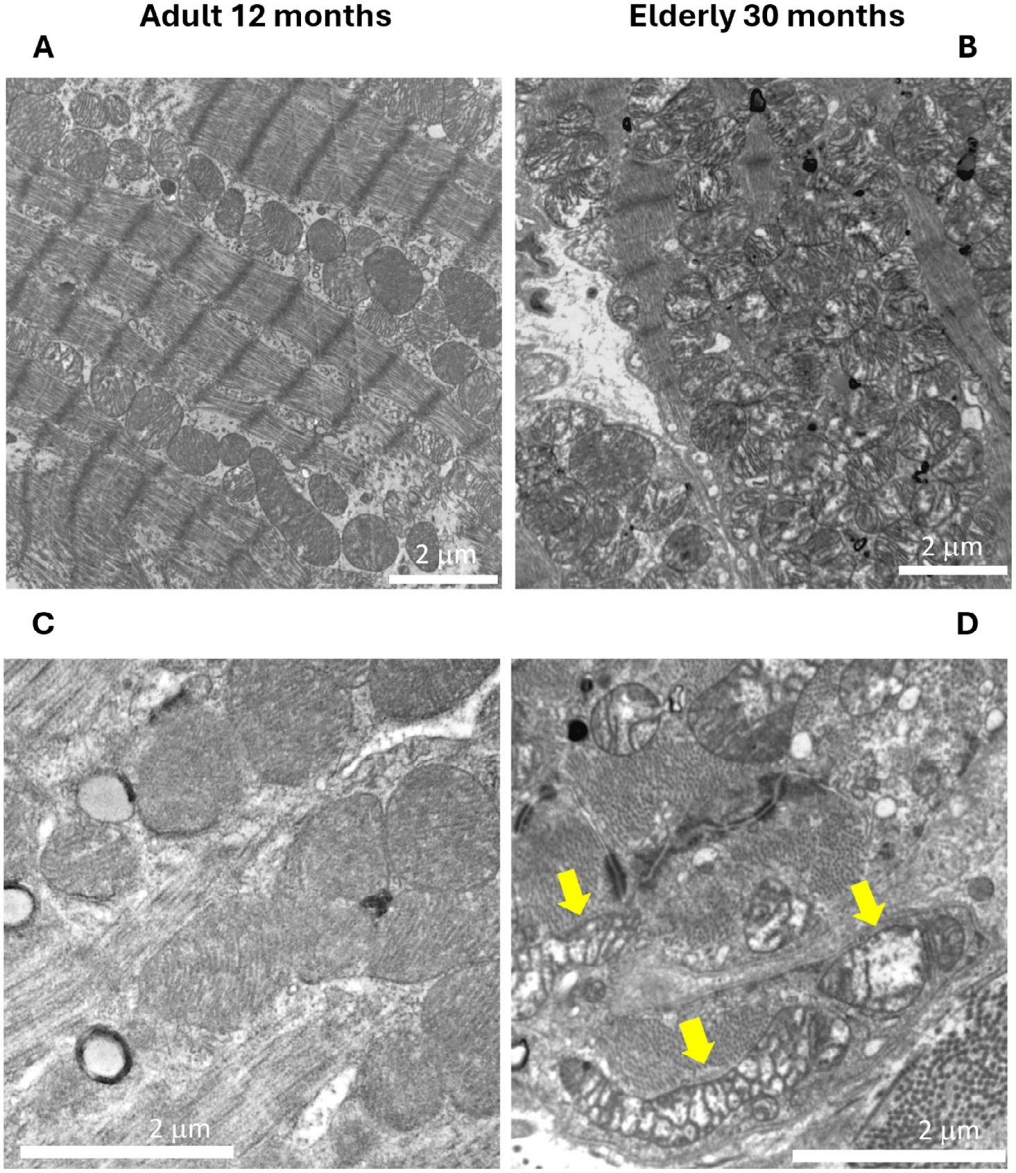
(A–D) Transmission electron microscopy (TEM) images illustrate mitochondrial ultrastructure in cardiomyocytes from adult (12‐month‐adult; A and C panels) and elderly mice (30‐month‐old; B and D panels). In adult hearts, mitochondria exhibit dense, well‐organized cristae and preserved matrix architecture. In contrast, aged cardiomyocytes display marked mitochondrial remodeling, with evident swelling, disrupted cristae morphology and reduced cristae density (highlighted by yellow arrows panel D) Scale bars = 2 μm.

**FIGURE 3:**
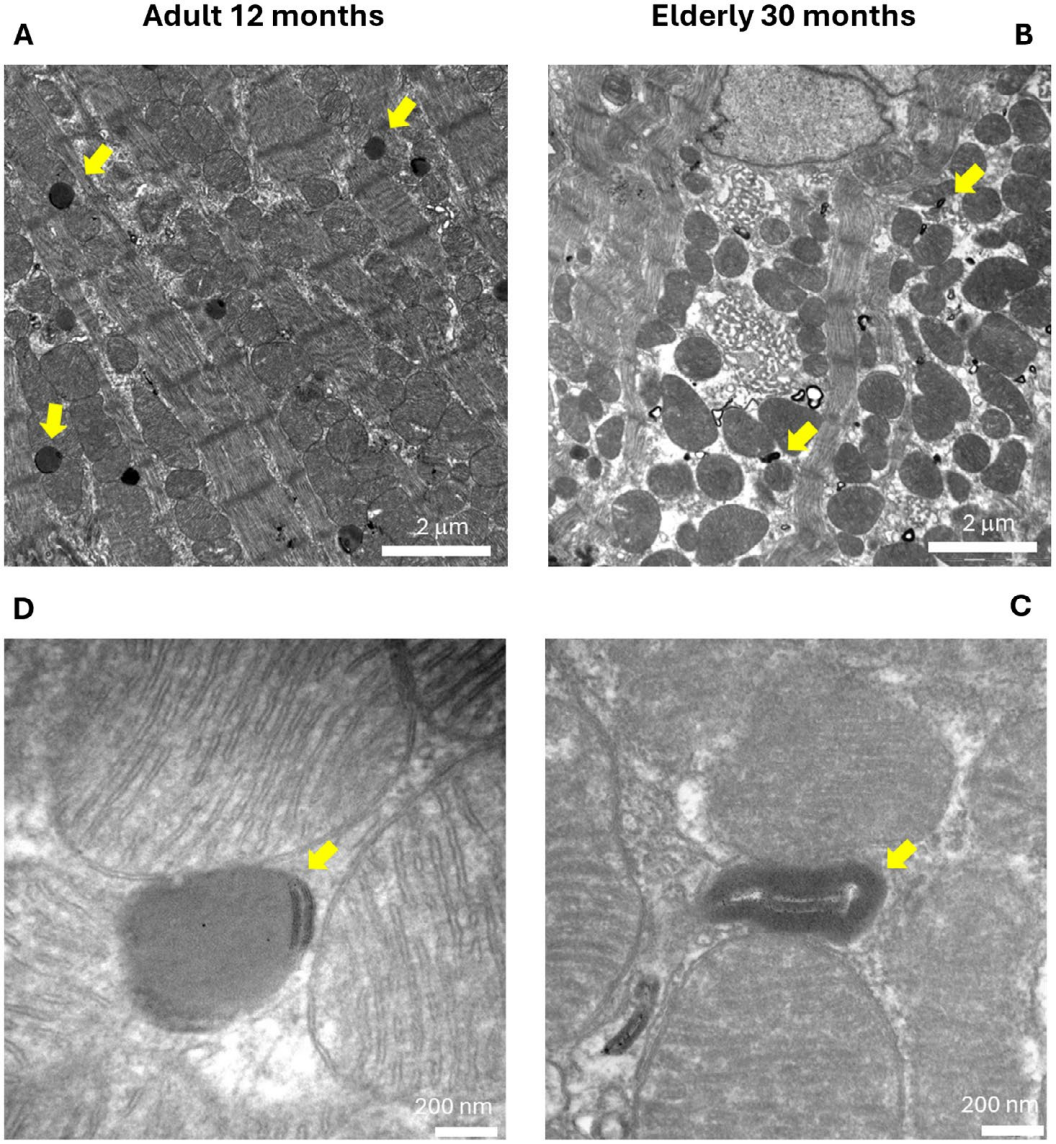
(A–D) Transmission electron microscopy (TEM) images comparing subcellular morphology in the hearts of adult (12 months; panels A and C) and elderly mice (30 months; panels B and D). (A, C) In the adult heart, lipid droplets (LDs) with canonical morphology (yellow arrows) are regularly distributed among the myofibrils. Mitochondria display normal morphology with well‐organized cristae. (B, D) In the elderly heart, normal lipid droplets become less frequent and are replaced by numerous concentric lamellar whorls (LWs; yellow arrows), indicative of depleted lipid content. Scale bars: 2 μm (A, B); 200 nm (C, D).

We evaluated the ultrastructure of the posterior wall of the left ventricle in the hearts of mice at different ages. The results reveal marked age‐related alterations mainly affecting mitochondria and lipid droplets. Early mitochondrial cristae rarefaction and dissolution were evident at 24 months. At 30 months, aged cardiomyocytes displayed pronounced mitochondrial remodeling, characterized by swollen mitochondria, disrupted cristae morphology, and reduced cristae density, frequently detected in multiple areas compared to adult hearts. (Figure 2A–D). A detailed morphometric analysis of the mitochondrial ultrastructure uncovered a pronounced and statistically significant difference in cristae score and the enlargement in the mitochondrial area which were positively correlated with older age. This enlargement may reflect compensatory adaptations to cellular or metabolic stress, despite the increase in size, circularity, area, cristae score, and solidity parameters (Figure S2A–C). The lipid droplets (LD) were also represented differently in elderly mice compared to adults. In the elderly heart, lipid droplets with canonical morphology (Figure 3A–C) were less frequent, whereas numerous lamellar whorls (LW), indicative of exhausted lipid content, were present (Figure 3B–D). These data indicate that age grouping correlated with the expected biochemical and clinical markers of aging.

### 3.3 Spatial‐RNA Seq Identifies Differentially Expressed Genes in the Aging Cardiomyocyte

**FIGURE 4:**
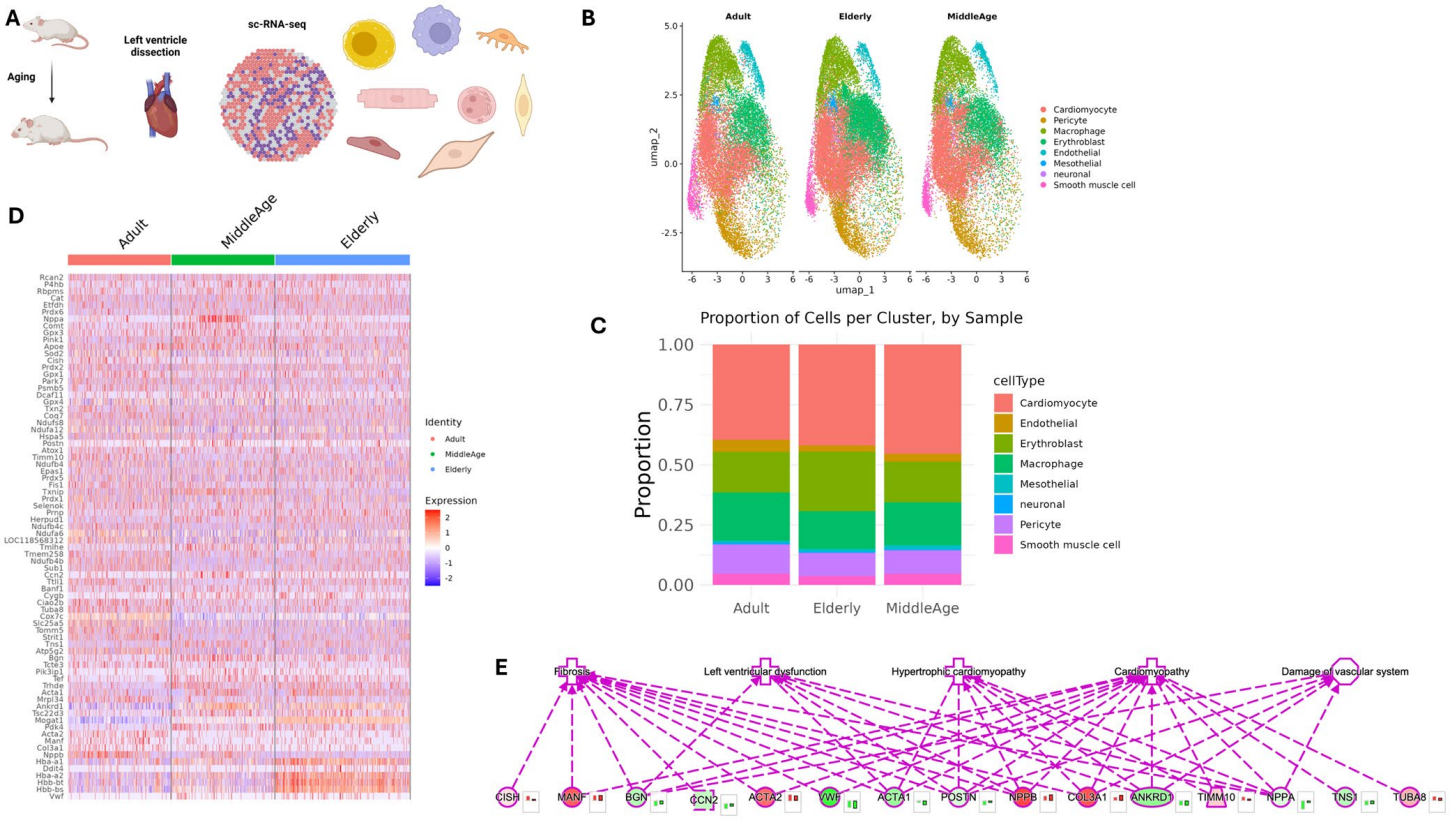
(A–E) (A) Schematic flowchart illustrating the workflow from left ventricle isolation followed by single‐cell RNA sequencing (scRNA‐seq) to assess transcriptomic changes. (B) UMAP (Uniform Manifold Approximation and Projection) visualization of the main cell types identified across in left ventricle slices of adult, middle age and elderly mice. Cells were clustered into 8 distinct subtypes, shown here with different colors. (C) Bar graph shows relative percentage of major cell types. (D) DEG heatmap showing relative trend of statistically significant genes. (E) Ingenuity Pathway Analysis using as background the terms “aging” and “heart with main terms highlighted and gene relative trend.

We used spatial transcriptomics to explore the cellular composition of the individual sections. We performed and analyzed single‐cell transcriptomic profiles from mouse left ventricles at different ages; the overall workflow is depicted in Figure 4A. As a result, unsupervised clustering scRNA‐seq analysis identified eight main cell subtypes, as illustrated on the UMAP plot (Figure 4B), encompassing cardiomyocytes, pericytes, macrophages, erythroblasts (endothelial cells, mesothelial cells, neuronal, and smooth muscle cells) (Enlargement reported in Figure S3). Cardiac spatial clusters were broadly consistent across the different aged samples, confirming that the characteristics of each cluster's distribution were similar and reproducible. As expected, cardiomyocytes were the dominant cell type (Figure 4C). Next, we performed differentially expressed gene (DEG) analysis of adult slides against middle‐aged and elderly samples. The heatmap reported in Figure 4D shows 76 DEGs. Performing pathway enrichment using the organism mouse and the terms “aging” and “heart” as a background through ingenuity pathway analysis (IPA), the most enriched terms were fibrosis, left ventricular dysfunction, hypertrophic cardiomyopathy, and, more generally, cardiomyopathy and damage to the vascular system (for all *p* values < 0.001) (Figure 4E). In the aged heart, in the network the following genes were downregulated and closely connected [Cytokine Inducible SH2 Containing Protein (CISH), Mesencephalic Astrocyte‐Derived Neurotrophic Factor (MANF), Actin Alpha 2, Smooth Muscle (ACTA2), Natriuretic Peptide B (NPPB), Collagen Type III Alpha 1 Chain (COL3A1), Translocase of Inner Mitochondrial Membrane 10 (TIMM10), and Tubulin Alpha 8 (TUBA8)]. In elderly mice, there was a parallel upregulation of the following genes [Biglycan (BGN), Cellular Communication Network Factor 2 (CCN2, also known as Connective Tissue Growth Factor, CTGF), Von Willebrand Factor (VWF), Actin Alpha 1, Skeletal Muscle (ACTA1), Periostin (POSTN), Ankyrin Repeat Domain 1 (ANKRD1), Natriuretic Peptide A (NPPA), Tensin 1 (TNS1)]. Collectively, spatial‐RNAseq provided a clear picture of the age‐related changes in gene expression toward a fibrotic phenotype.

### 3.4 Label‐Free Quantification (LFQ) Proteomics Analysis Highlights Key Proteins and Signaling Pathways in Heart Aging

**FIGURE 5:**
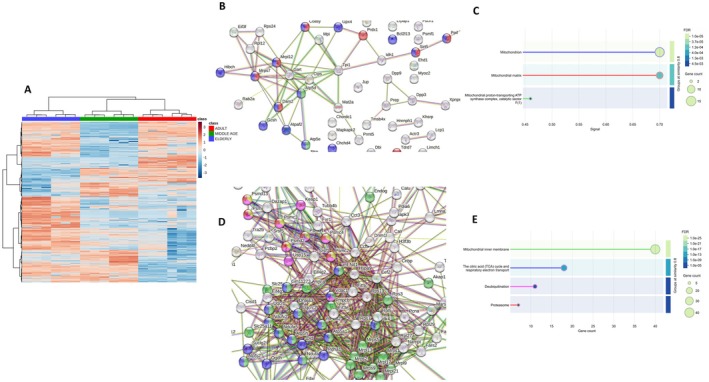
(A–E) Hierarchical clustering showing statistically significant proteins from ANOVA, (A) STRING network of downregulated proteins, (B), with corresponding enrichment (C), and STRING network of upregulated proteins, (D), with enrichment of main terms, (E).

LFQ proteomics resulted in over 1500 identified and relatively quantified proteins at FDR 1%, following alignment and filtering for missing values. Statistically significant proteins from ANOVA were employed to build a hierarchical clustering which revealed a net age‐dependent separation of the different groups, indicating deep proteome remodeling during aging (Figure 5A). The dataset was composed of 884 proteins (*p* < 0.05, FDR 0.05%, Table S2), which were utilized for STRING enrichment and Gene Ontology (GO) analysis. Such analyses revealed that over 80% of the dominant pathways (Figure S4) were represented by proteins involved in aging and mortality processes and mitochondrial membrane proteins (Figure S5), followed by abnormal myocardial fiber morphology, decreased ventricular muscle contractility, and cardiac fibrosis. In the downregulated protein cluster (Figure 5B), a crucial node was represented by mitochondrial matrix protein, such as Complex V, which is the final multi‐subunit complex of the OXPHOS system. Representative proteins were Atp5d and Atp5e (Figure 5C). They harness the proton electrochemical gradient energy to synthesize ATP from ADP and inorganic phosphate, representing the primary energy source for intracellular metabolic pathways. The reduction of these proteins pinpoints a failing mitochondrial status and a decreased ATP‐generating capacity, leading to increased reactive oxygen species (ROS). The concomitant decline in antioxidant defenses exacerbates the oxidative stress, namely, Glutathione peroxidase 4 (GPx4) and Peroxiredoxin 1 (Prdx1), ultimately increasing oxidative damage to mitochondrial proteins. Moreover, the downregulation of Peptidyl‐prolyl cis‐trans isomerase F (PPif) may contribute to altered mitochondrial permeability transition pore (mPTP) dynamics, further destabilizing mitochondrial function and promoting cell stress or death pathways. Additional age‐related evidence of mitochondrial reprogramming includes the upregulation of numerous proteins involved in substrate oxidation, the tricarboxylic acid cycle (TCA) and the respiratory electron transport chain, such as pyruvate dehydrogenase E1 (Pdha1), succinate‐CoA ligase (Suclg2), 2‐oxoglutarate dehydrogenase (Ogdh), dihydrolipoyl dehydrogenase (Dld), and NADH dehydrogenases (Ndufa5, Ndufa7, and Ndufv2). Such data suggest that glucose metabolism is reprogrammed in aged cardiomyocytes, with a significant increase in glycolysis (Serio et al. 2023). Furthermore, the increase of several proteins involved in the Ubiquitin‐Proteasome System, such as 26S proteasome components (Psmc2, Psmc3, Psmc4, Psmd2, Psmd4, and Psmd13) and Ubiquitin carboxyl‐terminal hydrolase 15 (Usp15) (Figure 5D,E), suggests a complex cellular adaptation to cardiac aging, characterized by mitochondrial dysfunction, compensatory responses, and structural remodeling (Hedhli and Depre 2010). Globally, proteomics reveals a profound and connected alteration of mitochondrial homeostasis toward impaired energy production.

### 3.5 Metabolomics and Lipidomics Reveal Oxidative Stress and Lipotoxicity

**FIGURE 6:**
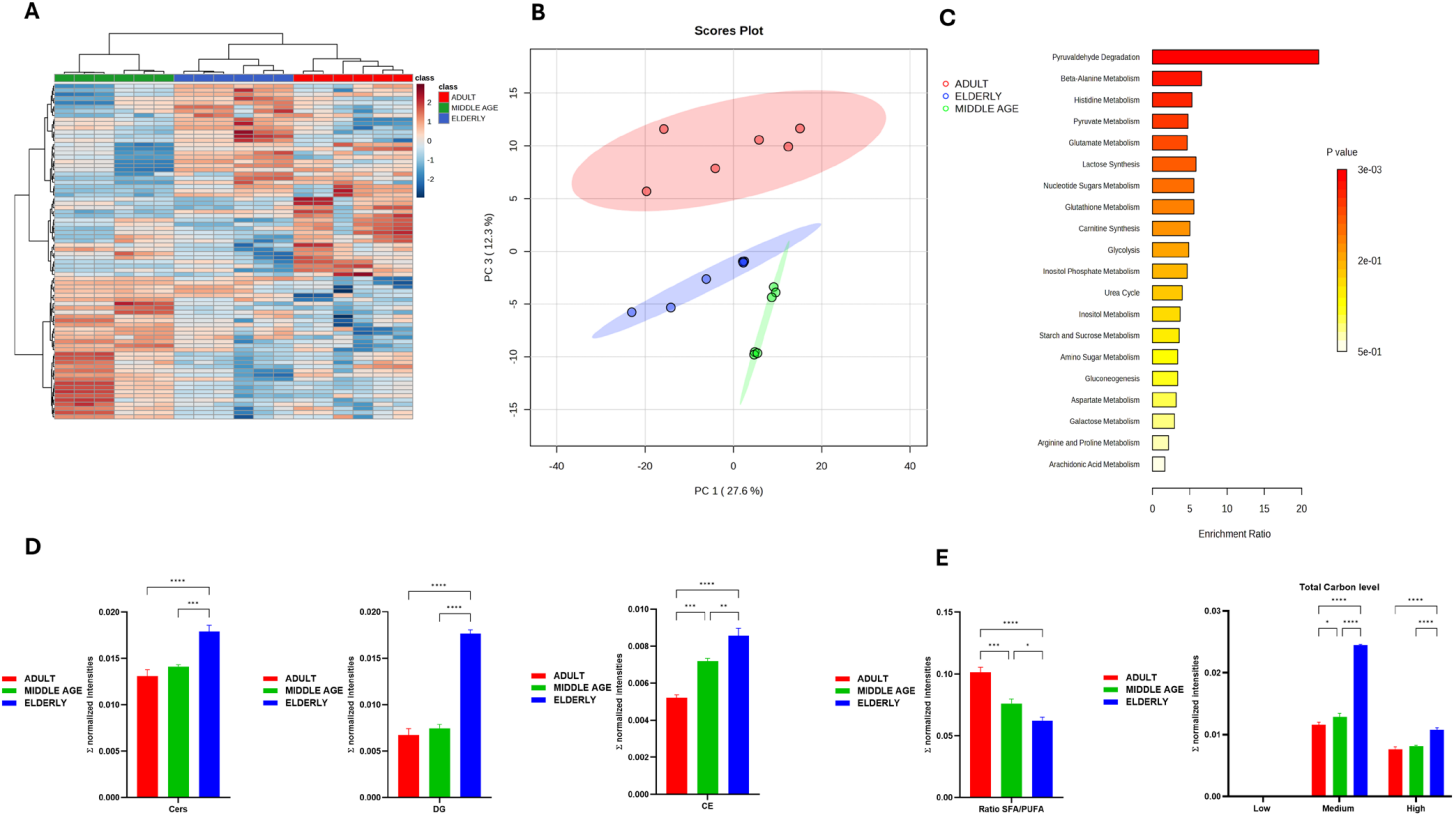
(A–D) (A) Hierarchical clustering showing the top 90 statistically significant metabolites from ANOVA expression across the aged heart; (B) SMPDB enrichment; (C) main lipid subclasses remodeling across the aged heart Ceramides (Cer), Diacylglycerols (DG), Cholesterol Esters (CE), (D) characteristics of double bonds and chain length for Cer and DG. Low: < 20 carbons, Medium: 20 < 40 carbons, High: > 40 carbons.

Metabolite profiling revealed a profound remodeling of both polar metabolites and lipid species, as clearly demonstrated by both hierarchical clustering and PCA score plot (Figure 6A,B), in which it can be observed how the three different age groups clearly cluster along PC1–PC3. Loading plots of significant metabolites are reported in Figure S6. Human Metabolome Database (HMDB) and Kyoto Encyclopedia of Genes and Genomes (KEGG) codes from statistically significant metabolites from ANOVA (Table S3) were used to perform pathway enrichment analysis on the basis of the Small Molecule Pathway Database (SMPDB), highlighting significant alterations in key metabolic routes (Figure 6C). Among the top enriched pathways (*p* < 0.05) were pyruvaldehyde (methylglyoxal) degradation, as well as alanine, histidine, and pyruvate metabolism—processes that are closely linked to cellular redox balance, glycation stress, and energy production. In the context of ventricular aging, these pathways are particularly relevant as they converge on mitochondrial function, a key determinant of cardiomyocyte homeostasis. Methylglyoxal degradation is critical in limiting the accumulation of advanced glycation end‐products (AGEs), which are known to impair mitochondrial enzymes and promote oxidative stress. Similarly, alanine, histidine, and pyruvate metabolism are central to mitochondrial bioenergetics, as these amino acids feed into the TCA cycle or influence NAD+/NADH balance. The age‐related metabolic changes resulted in increased levels of S‐LactoylGlutathione (LGSH), a key intermediate in the methylglyoxal detoxification pathway, alongside a progressive decline in reduced glutathione (GSH) and imidazole dipeptides such as carnosine and anserine. Elevated LGSH levels suggest an increased burden of methylglyoxal. This reactive dicarbonyl compound can modify proteins, lipids, and nucleic acids, forming AGEs that impair mitochondrial enzymes and contribute to ROS generation. The concomitant depletion of GSH, a major mitochondrial antioxidant, further compromises the cell's ability to neutralize oxidative damage, creating a vicious cycle of redox imbalance and bioenergetic failure. In parallel, the observed decline in carnosine and anserine, dipeptides with potent antioxidant and anti‐glycation properties, may further impair mitochondrial function and structural integrity, ultimately promoting cardiomyocyte dysfunction and age‐related ventricular remodeling (Anderson et al. 2018; Luengo et al. 2019). These findings suggest that mitochondrial dysfunction in the aged heart is structural and closely tied to specific metabolic shifts that compromise redox homeostasis and energy production, potentially contributing to age‐related ventricular remodeling and functional decline (Table 1). In the elderly group, lipidomic analysis revealed a marked accumulation of ceramides (Cers) and diacylglycerols (DGs), predominantly comprising medium‐ and long‐chain fatty acids (ranging from C20 to over C40), along with a higher degree of unsaturation compared to adult and middle‐aged hearts (Figure 6D,E, Table S4). This metabolite and lipid signature strongly indicates sustained mitochondrial stress and impaired redox buffering capacity in the aging heart, accompanied by altered fatty acid handling, potentially reflecting an imbalance between fatty acid uptake, storage, and mitochondrial oxidation.

**TABLE 1:** The table reports the metabolites significantly altered with aging, their direction of change in elderly versus adult animals, and their functional relevance. Aging was associated with increased lactoylglutathione (LGSH), ceramides (Cers), and diacylglycerols (DGs), together with decreased reduced glutathione (GSH), carnosine, and anserine, reflecting impaired antioxidant capacity, increased oxidative stress, and activation of lipotoxic and pro‐apoptotic pathways.

Metabolite	Trend with age	Functional relevance
Lactoylglutathione (LGSH)	↑ Elderly vs. Adult	Marker of glycolysis/oxidative stress
Reduced Glutathione (GSH)	↓ Elderly vs. Adult	Antioxidant depletion, impaired redox balance
Carnosine	↓ Elderly vs. Adult	ROS/RNS scavenger, loss of geroprotective role
Anserine	↓ Elderly vs. Adult	Antioxidant, anti‐glycation
Ceramides (Cers)	↑ Elderly vs. Adult	Lipotoxicity, pro‐apoptotic signaling
Diacylglycerols (DGs)	↑ Elderly vs. Adult	Lipid accumulation, mitochondrial impairment

## 4 Discussion

In the present study, we investigated mice of different ages to gain insights into the molecular mechanisms underlying cardiac aging. Combining data from electron microscopy imaging with gene expression, metabolomics, and protein profiling, we identified a complex metabolic remodeling, suggesting that, in mice, aging‐related cardiac impairment is primarily driven by mitochondrial dysfunction (Shao et al. 2020) (see Graphical abstract).

A distinct feature of our study was the CFI, which allowed us to categorize only the 30‐month‐old mice having a pronounced frailty phenotype, consistent with a geriatric stage of biological aging.

Interestingly, echocardiographic examination revealed a non‐significant, slight trend of age‐dependent decline in fractional shortening and ejection fraction, which is unlikely to have functional consequences. This finding supports the observation that diastolic rather than systolic dysfunction is the main consequence of cardiac aging. We used a combination of omics to study cardiac muscle to shed light on the key molecular and cellular processes that drive these functional consequences. This allowed us to correlate biological aging with molecular features of aging. ScRNA‐Seq approaches have evidenced that aged fibroblasts and upregulated proinflammatory response may impair microcirculation and perfusion, likely causing a discrepancy between oxygen and nutrient demand. This hypothesis is consistent with the findings of structural and functional mitochondrial abnormalities, such as widened cristae and impaired energy production in the heart of older mice (Brandt et al. 2017).

In particular, the EM imaging showed increased mitochondrial size coupled with well‐known structural evidence of mitochondrial dysfunction. The alteration observed in the mitochondrial cristae is significant because cristae morphology is essential to maintain the dense packing and architectural organization of the respiratory complexes, which is critical to the process of oxidative phosphorylation and ATP production, and to avoid the excessive production of ROS. Our observation corroborates the findings of previous studies reporting loss of the integrity and organization of mitochondrial cristae associated with oxidative stress, mitochondrial DNA damage, and disrupted lipid metabolism (Kondadi et al. 2019). The resulting increase of ROS further damages mitochondrial and cellular components in a vicious cycle. Such alterations in mitochondrial ridge structure and function contribute to a decline in cardiac performance, trigger inflammation, cause increased fibrosis, and a heightened risk of age‐related cardiovascular diseases. So far, a comprehensive multi‐omics analytical approach would helpfully elucidate the complex molecular processes behind the imaging findings.

ScRNA‐Seq analyses highlighted an age‐related downregulation of the *NPPB, MANF, CISH and TIMM10* genes, which are strictly related to age‐related cardiac functional dysfunction. Downregulation of the *NPPB* gene, encoding brain natriuretic peptide (Tamura et al. 2000) (BNP) prompts cardiac fibroblasts' proliferation and, in turn, impaired ATP with extracellular matrix deposition (Garvin and Hale 2022). *MANF* gene downregulation has also been associated with endoplasmic reticulum (ER) stress and altered mitochondrial homeostasis (Sousa‐Victor et al. 2019). Finally, *CISH* (a member of the *SOCS* suppressors of cytokine signaling) and *TIMM10* gene downregulation are associated with an excessive inflammatory response and defective assembly of respiratory chain complexes, respectively (Shoger et al. 2021). In contrast, the upregulation of multiple genes related to the remodeling profibrotic role of ECM structure and plasticity (*BGN, CCN2/CTGF*, *VWF*, A*CTA1*, *POSTN*, *ANKRD1*, *NPPA*, and *TNS1*) suggests that combined down and upregulation of mitochondrial genes have a double hit in age‐related mitochondrial dysfunction in cardiac aging. The changes in mitochondrial cristae architecture are reflected in the proteomic analyses. In particular, the reduction of Atp5d and Atp5e combined with the increase in the three‐carboxylic acid cycle (TCA) and respiratory electron transport of related proteins, suggests a reprogrammed metabolism toward a rise in glycolysis, which is a deficient cardiomyocyte's energetic tool (Zhou and Tian 2018). Supporting the hypothesis of a shift in energetic metabolism from oxidative phosphorylation to glycolysis, the metabolomic and lipidomic analyses evidenced the accumulation of LGSH, a biomarker of increased glucose metabolism and inflammatory conditions that frequently occur also in type 2 diabetes. Such a latter mechanism parallels an augmented oxidative stress by increased ROS levels, as highlighted by the downregulation of Gpxr4 and Prdx1 proteins (Cole‐Ezea et al. 2012). On the basis of these findings, one can conclude that as age progresses, cardiomyocytes work harder but with reduced energy efficiency. The resulting chronic discrepancy between energy production and utilization may lead to cardiac dysfunction, as previously reported in studies linking mitochondrial energetic imbalance with impaired cardiac performance (Zhou and Tian 2018; Xie et al. 2023). The metabolomic analysis confirms the notion that part of cardiac damage was due to excessive oxidative stress‐related cardiac damage as evidenced by the reduction in carnosine and anserine metabolites, both scavengers of ROS and reactive nitrogen species (RNS) and well‐known geroprotective molecules (Wang et al. 2024). Lipidomic analyses depicted the accumulation of Cers and DGs, which contribute to cardiomyocyte lipotoxicity and apoptosis (Xie et al. 2023). Cers and DGs activate pro‐apoptotic signaling pathways, promote oxidative stress, and impair electron transport chain activity. Their accumulation can, therefore, compromise mitochondrial ATP production and trigger cell death programs, contributing to age‐related degeneration. So far, the lipidomic alterations observed in aging hearts contribute to underlining that metabolic inflexibility and lipotoxic stress develop in aged cardiomyocytes, progressing toward diastolic ventricular dysfunction with aging (Actis Dato et al. 2022). Although our multi‐omics analysis identified several potential therapeutic targets, further validation will be required to confirm their functional relevance and therapeutic potential. Future studies using functional assays (e.g., knockdown or overexpression approaches) and pharmacological interventions will be essential to determine whether these targets can be effectively leveraged to mitigate cardiac aging.

## 5 Conclusions

Collectively, this multi‐omics approach delineates a complex interplay of multiple molecular mechanisms characterizing age‐related cardiac function. By integrating data across different biological layers, we have identified key pathways and molecules involved in the progression of cardiac dysfunction. Our findings highlight the central role of mitochondrial dysfunction and metabolic inflexibility in cardiac aging, suggesting that interventions aimed at preserving mitochondrial integrity and energy homeostasis may represent promising therapeutic strategies. Future studies should investigate the role of specific genes and signaling pathways in regulating mitochondrial function and assess whether targeting energy metabolism can mitigate age‐associated cardiac decline. Additionally, in vivo studies will be necessary to validate our results and confirm their relevance to cardiac aging.

## Author Contributions

Have made substantial contributions to conception and design, or acquisition of data, or analysis and interpretation of data: Manuela Giovanna Basilicata, Marco Malavolta, Serena Marcozzi, Gianluca Fulgenzi, Laura Graciotti, and Lucia Scisciola. Been involved in drafting the manuscript or revising it critically for important intellectual content: Manuela Giovanna Basilicata, Marco Malavolta, Eduardo Sommella, Fabrizio Merciai, Valentina Golino, and Tatiana Spadoni. Given the final approval of the version to be published. Each author should have participated sufficiently in the work to take public responsibility for appropriate portions of the content: Tania Ciaglia, Leonardo Schirone, Valentina Valenti, Sebastiano Sciarretta, and Giovanni Tortorella. Agreed to be accountable for all aspects of the work in ensuring that questions related to the accuracy or integrity of any part of the work are appropriately investigated and resolved: Ceereena Ubaida‐Mohien, Carmine Pizzi, Rafael De Cabo, Pietro Campiglia, Lucia Altucci, Michelangela Barbieri, Fabiola Olivieri, Luigi Ferrucci, and Giuseppe Paolisso.

## Conflicts of Interest

The authors declare no conflicts of interest.

## Supporting information


**Figure S1:** Electrocardiographic markers of diastolic function (PR interval, HRV) were used to calculate a Diastolic Dysfunction Index (DDI) in mice aged 12, 24, and 30 months. The DDI increased significantly with age, with 30‐month‐old mice showing the highest values, indicating advanced autonomic and electrical remodeling (one‐way ANOVA, *p* < 0.05). This index distinguished between early and late aging stages.
**Figure S2: (**A) Bar graph showing a significant increase in mitochondrial Area in the Middle age and Elderly groups compared to the Adult group (* = *p* < 0.05). (B) Histogram of mitochondrial size distribution. The *y*‐axis represents the frequency (number of mitochondria per size bin). The distribution shows a clear shift toward larger mitochondrial areas in the Middle age (red) and Elderly (blue) groups compared to the Adult group (green), indicating a trend toward increased mitochondrial size with aging. (C) Bar graph illustrating mitochondrial Circularity index across the three experimental groups. (D) Bar graph illustrating the mitochondrial Cristae score across the three experimental groups. A significant increase was detected in the elderly group compared with the adult (**p < 0.001) and middle‐aged (p* < 0.005) groups.
**Figure S3:** UMAP projection of single‐cell transcriptomes from adult, middle‐aged, and elderly hearts. Each dot represents a single cell, colored according to the annotated cell type. Major cardiac populations are highlighted, including cardiomyocytes, pericytes, macrophages, erythroblasts, endothelial cells, mesothelial cells, neurons, and smooth muscle cells. The distribution across age groups illustrates the preservation of major cell identities with age, while allowing the assessment of subtle shifts in cellular composition and transcriptomic remodeling.
**Figure S4:** String protein–protein network of significant proteins from Anova, colored nodes belong to different enriched pathways; please see Figure 3.
**Figure S5:** Most enriched pathways related to the string network shown in Figure S4.
**Figure S6:** Loading plot with explicit metabolite labels shows the distribution of metabolites along the first two loading components (Loadings 1 and Loadings 2). Each point represents a metabolite, with position indicating its relative contribution to the model.
**Table S1:** Standard echocardiographic parameters in mice at 6, 12, 24, and 30 months of age, including heart rate (HR), end‐diastolic diameter (EDD), end‐systolic diameter (ESD), fractional shortening (FS), ejection fraction (EF), and cardiac output (CO).
**Table S2–S4:** Anova results from proteomics (S2), metabolomics (S3) and lipidomics (S4) reporting Unprot ID and metabolite‐lipid annotation, respectively.

## Data Availability

All data associated with this manuscript have been uploaded to the repository Zenodo (https://zenodo.org/) with doi:10.5281/zenodo.15275340; additional data are available upon request.
