# Supplementary material for: Electron Microscopy and Multi‐Omics Reveal Mitochondrial Dysfunction and Structural Remodeling in the Hearts of Elderly Mice
Source: Aging Cell. 2025 Nov 18;24(12):e70286. doi: 10.1111/acel.70286 (PMC12686548; doi:10.1111/acel.70286)
Supplement: Supplementary file 1 — Figure S1: Electrocardiographic markers of diastolic function (PR interval, HRV) were used to calculate a Diastolic Dysfunction Index (DDI) in mice aged 12, 24, and 30 months. The DDI increased significantly with age, with 30‐month‐old mice showing the highest values, indicating advanced autonomic and electrical remodeling (one‐way ANOVA, p < 0.05). This index distinguished between early and late aging stages. Figure S2: (A) Bar graph showing a significant increase in mitochondrial Area in the Middle age and Elderly groups compared to the Adult group (* = p < 0.05). (B) Histogram of mitochondrial size distribution. The y‐axis represents the frequency (number of mitochondria per size bin). The distribution shows a clear shift toward larger mitochondrial areas in the Middle age (red) and Elderly (blue) groups compared to the Adult group (green), indicating a trend toward increased mitochondrial size with aging. (C) Bar graph illustrating mitochondrial Circularity index across the three experimental groups. (D) Bar graph illustrating the mitochondrial Cristae score across the three experimental groups. A significant increase was detected in the elderly group compared with the adult (**p < 0.001) and middle‐aged (p* < 0.005) groups. Figure S3: UMAP projection of single‐cell transcriptomes from adult, middle‐aged, and elderly hearts. Each dot represents a single cell, colored according to the annotated cell type. Major cardiac populations are highlighted, including cardiomyocytes, pericytes, macrophages, erythroblasts, endothelial cells, mesothelial cells, neurons, and smooth muscle cells. The distribution across age groups illustrates the preservation of major cell identities with age, while allowing the assessment of subtle shifts in cellular composition and transcriptomic remodeling. Figure S4: String protein–protein network of significant proteins from Anova, colored nodes belong to different enriched pathways; please see Figure 3. Figure S5: Most enriched pathways r [file ACEL-24-e70286-s001.docx]

**Electron microscopy and multi-omics reveal mitochondrial dysfunction and structural remodeling in the hearts of elderly mice**

Manuela Giovanna Basilicata^1^*****, Marco Malavolta^2,3^*****, Serena Marcozzi^2,4^, Eduardo Sommella^5^, Lucia Scisciola^1^, Fabrizio Merciai^5^, Gianluca Fulgenzi^2,3^, Valentina Golino^5,6^, Giovanni Tortorella^1^, Tatiana Spadoni^7^, Laura Graciotti^7^, Tania Ciaglia^5^, Leonardo Schirone^8^, Valentina Valenti^9^, Sebastiano Sciarretta^9,10^, Ceereena Ubaida-Mohien^11^, Carmine Pizzi^12,13^, Rafael De Cabo^14^, Pietro Campiglia^5^, Lucia Altucci^15,16,17^, Michelangela Barbieri^1^, Fabiola Olivieri^2,3^, Luigi Ferrucci^14^ and Giuseppe Paolisso ^1,18^

^1^Department of Advanced Medical and Surgical Sciences, University of Campania “Luigi Vanvitelli”, Naples, Italy.

^2^ Advanced Technology Center for Aging Research and Geriatric Mouse Clinic, IRCCS INRCA, 60121 Ancona, Italy.

^3^ Department of Clinical and Molecular Sciences (DISCLIMO), Polytechnic University of Marche, Ancona, Italy.

^4^ Scientific Direction, IRCCS INRCA, 60124 Ancona, Italy.

^5^ Department of Pharmacy, University of Salerno, Fisciano, SA, Italy.

^6^ National PhD Program in RNA Therapeutics and Gene Therapy, University of Naples Federico II, 80131 Napoli, Italy.

^7^ Department of Biomedical Sciences and Public Health, Polytechnic University of Marche, Ancona, Italy.

^8^ Department of Health and Life Science, European University of Rome.

^9^ Department of Medico-Surgical Sciences and Biotechnologies, University of Sapienza, Rome, Italy

^10^ IRCCS Neuromed, Pozzilli, Italy.

^11^ Intramural Research Program, National Institute on Aging, National Institutes of Health, Baltimore, Maryland, USA.

^12^ Department of Medical and Surgical Sciences-DIMEC-Alma Mater Studiorum, University of Bologna, Bologna, Italy.

^13^ Cardiovascular Division, Morgagni–Pierantoni University Hospital, Forlì, Italy.

^14^ National Institute on Aging, Baltimore, MD, USA.

^15^ Department of Precision Medicine University of Campania “Luigi Vanvitelli”, Naples, Italy

^16^ Program of Medical Epigenetics, Vanvitelli Hospital, Naples, 80138, Italy.

^17^ Biogem, Molecular Biology and Genetics Research Institute, Ariano Irpino, 83031, Italy.

^18^ UniCamillus International University of Health Sciences, 00161 Rome, Italy.

*** These authors equally contributed as first author**

**Corresponding Author**:

**Prof. Giuseppe Paolisso**

Department of Advanced Medical and Surgical Sciences, University of Campania 'Luigi Vanvitelli', Naples, Italy

e-mail: [giuseppe.paolisso@unicampania.it](mailto:giuseppe.paolisso@unicampania.it)

Tel: ++39.0815665135

**Supplementary Material**

**Supplementary Methods**

**Animals and Experimental Design**

Male C57BL/6J mice aged 6 months (young group, n = 9), 12 months (adult group, n = 12), 24 months (middle age group, n = 11), and 30 months (elderly group, n = 11) were enrolled in this study. All mice were bred and maintained at the Geriatric Mouse Clinic of IRCCS INRCA under SPF conditions. We included only male mice in this study to reduce variability related to hormonal cycles, which may influence cardiac physiology and mitochondrial function. That excluding female represents a limitation of the present work, as sex differences are known to affect cardiac aging, mitochondrial remodeling, and fibrotic pathways. Future studies including both sexes will be critical to determine whether the observed phenotypes are consistent across sexes or whether sex-specific trajectories of cardiac aging exist.

All the experimental protocols were approved by the Italian Ministry of Health (authorization n. 392/2019-PR). Mice were housed in SPF conditions at a maximum density of 4 per cage under a controlled environment, maintained on a 12:12 light-dark cycle (lights on at 6:00 a.m.), and had ad libitum access to food and water.

**Biochemical parameters**

Biochemical parameters were analyzed in a subset of animals in each age group: young (n = 6), adult (n = 6), middle age (n = 6), and elderly (n = 6). Blood samples were collected from the right retroorbital plexus of anesthetized mice. Anesthesia was induced by placing each mouse in an inhalation chamber with 4% isoflurane regulated with a calibrated vaporizer. Blood samples were collected, transferred into serum separator gel tubes (Microvette® CB 300 EDTA K2E tubes, cod.16.444.100, Sarstedt) and centrifuged for serum separation. Serum concentrations of glucose, albumin, and creatinine were determined using their appropriate kits (cod. CL35-400S, CL04-400S, and HP57-240 by Far Diagnostics, Italy, respectively) by an automated analyzer (COBAS MIRA, by Roche) according to the manufacturer’s instructions. Standard controls were run before each determination.

**Echocardiography and electrocardiography (ECG) analysis**

Echocardiography analysis was performed in a subset of animals in each age group: young (n = 6), adult (n = 9), middle age (n = 8), and elderly (n = 8) authorization n. 392/2019-PR and n. 59/2024-PR. Briefly, a high-frequency, high-resolution digital imaging platform with linear array technology and color Doppler mode for in vivo high-resolution micro-imaging was used (Vevo® 3100 Imaging System, FUJIFILM VisualSonics Inc., Toronto, Canada). To assess the cardiovascular function of mice, a high-frequency transducer probe (VisualSonics MS400, FUJIFILM VisualSonics, Inc., Toronto, Canada with a frequency range of 18–38 MHz) was used by a skilled cardiologist under the supervision of a veterinarian. Mice were anesthetized using (IsoFlo®) 1.35% + 2% O2, shaved and positioned on an electrically warmed surface.

ECG was recorded non-invasively in non-anesthetized, conscious mice using the ECGenie recording platform (Mouse Specifics, Inc.). All mice were allowed to acclimate for ~10 minutes. After the acclimation period, ECG signals were recorded while the mice passively established contact between the underside of their paws and the electrodes. Only data from continuous recordings of > 15 ECG signals were used in the analyses. Analysis of individual ECG signals was then performed using e-MOUSE physiologic waveform analysis software (Mouse Specifics, Inc.).

**Electron Microscopy data**

Heart was rapidly excised from avertin anesthetized mice. A cubic millimeter of posterior wall was dissected from the fresh tissue and submerged for 30 seconds in a myorelaxant solution (Phosphate buffer added with 50 mM of KCl) and then transferred to the fixative solution (2.5% glutaraldehyde, 0.5% paraformaldehyde, 0.7% tannic acid and 30 mM sucrose in 0.1 M of cacodylate buffer (pH 7.0) for 1 h at room temperature and after that stored in fixative at 4 C Until further processing. Hearth blocks were post fixed in 0.5% Osmium tetroxide in cacodylate buffer for 1 h then washed in distilled H2O. Blocks were dehydrated in acetone series and embedded in Epon-Araldyte resin. 50 nm thick section were obtained with Leica ultramicrotome (UM6 Leica, Germany) placed in 40 mesh copper grids, stained with lead citrate and imaged with a Philips CM12 electron microscope at 100 Kv.

Images at nominal magnification of 8800 x and resolution of 90.43 pix/µm were used to measure mitochondria. A multi-point spline was manually drawn to contour healthy mitochondria using ImageJ software, then the area, perimeter, minor axes, major axes, solidity, and circularity index, cristae score and mithocondrial number measured automatically by the software. To evaluate group differences while accounting for repeated measurements within individual animals, we employed Generalized Estimating Equations (GEE). Area and other variables measurements were collected in multiple replicates per mouse (100-300 measurements per animal) across three experimental groups (adult n = 3, middle age n = 2, and elderly n = 2). GEE models were fitted using a gamma distribution with a log link function to accommodate the positive skew and heteroscedasticity of the data. The mouse identifier (Mouse_ID) was included as the subject variable to adjust for intra-animal correlation. Estimated marginal means (EMMs) were computed for each group, and pairwise comparisons were performed using least significant difference (LSD) adjustment. All analyses were conducted using SPSS (Version 26, IBM Corp.).

**Statistical analysis for Animal Design**

Results were presented as mean ± SEM and p-value was determined by one-way ANOVA followed by Bonferroni post-hoc analyses using IBM SPSS Statistics version 29.0. The graphs were created using Graphpad Prism 9 software. A p value < 0.05 was used to determine significance for all statistical tests. * p < 0.05; ** p < 0.01; *** p < 0.001; **** p < 0.0001.

**Spatial transcriptomics**

Spatial transcriptomics was performed in a subset of animals in the groups: adult (n = 2), middle age (n = 2), and elderly (n = 2). Left ventricles were dissected, embedded in OCT, snap-frozen on dry ice and stored under liquid nitrogen.

Spatial transcriptomics was performed using BMKMANU S1000 RNA-seq with the BMKMANU S1000 Gene Expression kit (BMKGENE, ST03002) at BMKGENEMANU. Briefly, Spatial Transcriptomics slides were printed with 1-8 identical 6.8×6.8 mm capture areas, each with 2,000,000 spots contain barcoded primers (BMKMANU S1000). The primers are attached to the slide by the 5’ end and contain a cleavage site, a T7 promoter region, a partial read1 Illumina handle, a spot-unique spatial barcode, a unique molecular identifier (UMI), and Poly(dT)VN. The spots have a diameter of 2.5μm and are arranged in a centered regular hexagonal grid so that each spot has six surrounding spots with a center-to-center distance of 4.8μm. Tissue sectioning, blue staining, imaging, and first permeabilization were consistent with the user guide of the BMKMANU S1000 Tissue Optimization Kit (BMKMANU, ST03003). Secondary permeabilization (9 min) and library construction were also performed according to the user guide (supplementary material section s1). Reverse transcription (RT), second-strand cDNA synthesis, adaptor ligation and a second RT was generated, and libraries were constructed according to the performer’s protocol. Sequencing handles and indexes were added in an indexing PCR and the finished libraries were purified and quantified. Sequencing was performed on the Illumina NovaSeq 6000 with a sequencing depth of at least 50,000 reads per spot (100μm) and 150bp (PE150) paired end reads (performed by Biomarker Technologies Corporation, Beijing, China). We completed the upstream analysis through BSTMatrix (v1.0). The mapping was performed to the reference GRCh38_release95 human genome. The count matrixes data and the image adjusted by BSTViewer V4.4.8.1 and corresponding matrix were used for downstream analysis. Briefly, quality control and data analysis were carried out through steps of normalization, clustering and screening marker genes using R package Seurat (v5.1.0), followed by marker gene annotation. Visualization including UMAP and clustering was mainly done through R package using custom the R script. For the library construction sectioned slides were incubated at 37℃ for 1 min., fixed in 3.7%–3.8% formaldehyde (Sigma-Aldrich) in PBS (Medicago) for 30 min, and then washed in 1x PBS (Medicago). For staining, sections were incubated in Mayer’s hematoxylin (Dako, Agilent, Santa Clara, CA) for 4 min, bluing buffer (Dako) for 30 s, and Eosin (Sigma-Aldrich) diluted 1:5 in Tris-base (0.45M Tris, 0.5M acetic acid, pH 6.0) for 30 s. The slides were washed in RNase and DNase free water after each of the staining steps. After air-drying, the slides were mounted with 85% glycerol (Merck Millipore, Burlington, MA) and coverslips (Menzel-Glaser). Bright-field (BF) images were taken at 20x magnification using Metafer Slide Scanning platform (MetaSystems). Raw images were stitched with VSlide software (MetaSystems). The coverslip and glycerol were removed after imaging by immersing slides in RNase and DNase free water. The slides were inserted into slide cassettes to separate the tissue sections into individual reaction chambers (hereinafter wells). For pre-permeabilization, sections were incubated at 37℃ for 20 min with 0.5 U/ml collagenase (ThermoFisher) and 0.2 mg/ml BSA (NEB, Ipswich, MA) in HBSS buffer (ThermoFisher). Wells were washed with 0.1× SSC(Sigma-Aldrich), after which permeabilization was conducted at 37℃ for 7 min in 0.1% pepsin (Sigma-Aldrich) dissolved in 0.1M HCl (Sigma-Aldrich). After incubation, the pepsin solution was removed and wells washed with 0.1 × SSC.

**Proteomic Sample Preparation and Analysis**

Proteomics was performed in a subset of animals in the groups: adult (n = 2), middle age (n = 2), and elderly (n = 2). Chemicals LC–MS-grade Water (H2O) acetonitrile (ACN), methanol (CH3OH), isopropanol (IPA), 1-butanol (BuOH), methyl tert-butyl ether (MTBE), and additives formic acid (HCOOH), acetic acid (CH3COOH), ammonium formate (HCOONH4) and ammonium acetate (CH3COONH4), were all purchased from VWR (Milan, Italy). Deuterated and authentic lipid standards were purchased by Avanti Polar Lipids (Alabaster, AL, USA). Unless stated otherwise, other reagents were all purchased by Merck.

3 mg of cardiac ventricular tissues was lyophilized for 72 hours and subsequently homogenized through two 30-second steps at 500 g using a HT Lysing Homogenizer (OHAUS, Nänikon, Switzerland), protein content quantification was performed by BCA method (Thermo Fisher Scientific, Milan, Italy). Then iST (Preomics, Martinsried, Germany) sample preparation kit which includes all chemicals to denature, reduce and alkylate proteins, as well as the enzymes to perform a tryptic digestion and a final peptide cleanup was used according to the manufacturer’s instructions. Proteomic analysis was performed by nLC- HRMS using an Ultimate 3000 nanoLC (Thermo Fisher Scientific, Bremen, Germany) coupled to an Orbitrap Lumos tribrid mass spectrometer (Thermo Fisher Scientific) with an Easy nano electrospray ion source (Thermo Fisher Scientific). Peptides were trapped for 1 minute in a PepMap trap-Cartridge, 100 Å, 5 µm, 0.3 x 5 mm (Thermo Fisher), and separated onto a C18-reversed phase column (250 mm × 75 μm I.D, 2.0 µm, 100Å, Thermo). Mobile phases were A): 0,1% HCOOH in water v/v; B): 0,1% HCOOH in ACN/Water v/v 80/20. Peptides were separated using a linear gradient of 90 min. HRMS analysis was performed in data dependent acquisition (DDA), with MS1 range 400–1500 m/z, HCD fragmentation was used with normalized collision energy setting 27. Resolution was set at 120.000 for MS1 and 15.000 for MS/MS. Single charge and unassigned charge peptides were excluded. Quadrupole isolation was set to 3Da. Maximum ion injection times for MS (OT) and the MS/MS (OT) scans were set to auto and 50 ms respectively, and ACG values were set to standard. Dynamic exclusion: 30 s. For data processing, raw MS data were analysed using Proteome Discoverer v 2.5 (Thermo Fisher). The following parameters were used: enzyme trypsin, missed cleavages max 1, mass accuracy tolerance 10 ppm and 0,6 Da for precursors and fragments respectively. Sequest search and Percolator algorithm were used. Carbamidomethylcysteine was used as fixed modification while methionine oxidation as variable. Proteins were considered identified with at least one unique peptide, using a false discovery rate (FDR) thresholds of 0.01 (strict) and 1% (relaxed). Each analysis was performed in triplicate. For Proteome data processing, raw MS data were analysed using Proteome Discoverer v 2.5 (Thermo Fisher). The following parameters were used: enzyme trypsin, missed cleavages max 1, mass accuracy tolerance 10 ppm and 0,6 Da for precursors and fragments respectively. Sequest search Percolator and Inferys rescoring algorithm nodes were used. Carbamidomethylcysteine was used as fixed modification while methionine oxidation as variable. Proteins were considered identified with at least one unique peptide, using a false discovery rate (FDR) thresholds of 0.01 (strict) and 1% (relaxed). Each analysis was performed in triplicate. Proteomics Enrichment was performed by STRING (Szklarczyk et al. Nucleic Acids Res. 2015 43(Database issue): D447-52) significant proteins ID were uploaded in the multiple protein nodes against Mus Musculus background proteome, extension in Skyline (https://skyline.ms/project/home/begin.view) was used for in silico comparison of specific product ions.

**Metabolome and lipidome Sample Preparation and Analysis**

1 mg of lyophilized ventricle tissues was added to 100 µL of ice-cold MeOH/H2O (1:1 v/v %). Samples were shaken in a thermomixer (Eppendorf), put in an ultrasound bath, and subsequently centrifuged for 20 min at 4 °C and 20,238 rcf. Supernatants were discarded and pellets were extracted with 100 µL of MTBE containing a mix of deuterated standard and then, treated as previously described. Supernatants were dried using a SpeedVac (Savant, Thermo Scientific, Milan, Italy). Metabolome analyses were performed on a Thermo Vanquish Flex UHPLC system coupled online to a Exploris 120 hybrid quadrupole Orbitrap mass spectrometer (Thermo Fisher Scientific, Bremen, Germany) equipped with a heated electrospray ionization probe (HESI II). Lipidome analysis was performed on an Ultimate RS 3000 UHPLC (Thermo Fisher), coupled online to a TimsTOF Pro Quadrupole Time of Flight (Q-TOF) (Bruker Daltonics, Bremen, Germany) equipped with an Apollo II electrospray ionization (ESI) probe.

**Metabolomics and lipidomics analyses**

Metabo-lipidomics was performed in a subset of animals in the groups: adult (n = 2), middle age (n = 2), and elderly (n = 2). Metabolomics analyses were performed on a Vanquish Flex UHPLC system coupled online to an Exploris 120 hybrid quadrupole Orbitrap MS (Thermo Fisher Scientific, Bremen, Germany). In detail, metabolome analyses were performed in HILIC mode, separation was carried out with a BEH Amide column (100 × 2.1 mm; 1.7μm) protected with a Vanguard precolumn (5 × 2.1 mm; 1.7 μm) (Waters, Milan, Italy). The column temperature was set at 45 °C, and the flow rate was 0.400 mL/min. The mobile phase was (A): H2O/ACN 95/5 (v/v %) and (B): in H2O/ACN 5/ 95 (v/v %) both buffered with 10 mM CH3COONH4. The following gradient was employed: 0–0.1 min, 99 % B; 0.1–8 min, 99–50 % B; 8.0–8.5 min, 50-30 % B; 8.5-9.5 min isocratic at 30 % B; returning to 99% in 0.1 min, and then 4 min to recondition the column. the HESI source parameters were sheath gas pressure, 40 au. and 50 a.u. for positive and negative ionization mode, respectively; aux gas flow 15 a.u.; sweep gas flow, 0 a.u. Spray voltages were set to 3.3 kV and 3.0 kV for ESI (+) and ESI (-), Ion Transfer Tube (ITT) and Vaporizer temperature were set to 300 °C and 320 °C. MS data acquisition was performed in full scan-data dependent acquisition (FS-DDA) in the m/z 70-800, MS1 resolution was set to 60000, the AGC target was set to auto with a maximum injection time at 100 ms. MS/MS was employed with an isolation window of 1-5 Da, dynamic exclusion of 10s, resolution was set to 15.000, and HCD was used with normalized collision energies of 20, 40 and 60. The instrument was externally calibrated daily with FlexMix solution (Thermo Fisher) while at the beginning of every LC run the internal calibrant was injected (IC run start mode).

Lipidome analyses were performed on a Thermo Ultimate 3000 UHPLC coupled online with a quadrupole-time of flight MS TimsTof Pro (Bruker Daltonics, Bremen, Germany) equipped with an Apollo II ESI source. Lipid separation was performed with an Acquity UPLC CSHTM C18 column (50 × 2.1 mm; 1.7 μm, 130 Å) protected with a VanGuard CSHTM precolumn (5.0 × 2.1 mm; 1.7 μm, 130 Å) (Waters, Milford, MA, U.S.A). Column oven temperature was set at 65°C, flow rate was set to 0.55 mL/min, mobile phases composition was: (A): ACN/H2O 60:40 (v/v %) and (B): IPA/ACN 90:10 (v/v %) both buffered with 10 mM HCOONH4 and 0.1% HCOOH (v/v %), the following gradient has been employed: 0 min, 40% B; 0.4 min, 43% B; 0.425 min, 50% B; 0.9 min, 57% B; 2.0 min, 70% B; 2.950 min, 99% B; 3.3 min, 99% B; 3.31, 40% B and then 0.7 min for column re-equilibration. At the begininning of each run, the mass accuracy and mobility were recalibrated by injection of a mixture (1:1 v/v %) of 10 mM sodium formate calibrant solution and ESI-L Low Concentration Tuning Mix. Data-dependent parallel accumulation serial fragmentation (DDA-PASEF) acquisition mode was used. Both positive and negative ESI ionization were used, in separate runs, each sample was run in triplicate, 2 µL were injected. ESI source parameters: Nebulizer gas (N2) pressure: 4.0 Bar, Dry gas (N2): 10 L/min, Dry temperature: 280°C. Mass spectra were acquired in the range m/z 50–1500, with an accumulation and ramp time to 100 ms each. The ion mobility was scanned from 0.55 to 1.80 Vs/cm2. Precursors were isolated within ± 2 m/z and fragmented with an ion mobility-dependent collision energy ranging from 20 to 40 eV. The total acquisition cycle was of 0.32 s and comprised one full TIMS-MS scan and two PASEF MS/MS scans. Exclusion time was set to 0.1 min, Ion charge control (ICC) was set to 7.5 Mio. Additionally, a number of separate experiments was performed with different collision energy settings (TIMS-STEPPING: CE [eV] #1: 20-40 and CE [eV] #2: 35-50). Metabolomics analysis was performed by Compound Discoverer v.3.3 (Thermo Fisher Scientific) to normalize, align, detect and identify compounds. Features were extracted from 0-10 min and 0-11 min of the HILIC run, respectively, in the m/z=70-800 mass range. Data were aligned according to an adaptive curve alignment model. Compounds were detected using the following parameters settings: mass tolerance was set to 5 ppm, while retention time tolerance was set to 0.2 min; minimum peak intensity was set to 100000 AU and the signal-to-noise threshold for compound detection was set to 5. The peak rating filter was set to 3. To perform blank subtraction, we maintained max sample/max blank > 5. To predict elemental compositions of the compounds, the relative intensity tolerance was set to 30% for isotope pattern matching. For the mzCloud database search, both the precursor and fragment mass tolerance were set to 5 ppm. The databases used for matching compounds in ChemSpider for structural search were BioCyc, the Human Metabolome Database and KEGG, and the mass tolerance in ChemSpider Search was set to 5 ppm. The mass tolerance for matching compounds in Metabolika pathways was set to 5 ppm. Compounds were assigned by comparing annotations using the following nodes in order of priority: (1) mzCloud (2) Predicted Compositions; (3) MassList search; (4) ChemSpider Search; (5) Metabolika search. MetaboScape 2023b (Bruker) was used for lipidomics data processing and lipid annotation. Feature detection was set to 500 and 250 counts for positive and negative modes. The minimum number of data points was set to 100, and recursive feature extraction was used (75 points). Lipid annotation was first performed with a rule-based annotation, based on diagnostic-class specific fragments and their intensity in corresponding MS/MS spectra, and, subsequently, using the LipidBlast spectral library (http://prime.psc.riken.jp/compms/msdial/main.html). The following parameters were set Mass accuracy window: narrow 2 ppm, wide 10 ppm; mSigma: narrow 30, wide 250, MS/MS score: narrow 800, wide 150. Collision cross-section (CCS) %: narrow 1, wide 3.5. The spectra were processed in ESI+ using as adducts [M+H]+, [M+Na]+, [M+K]+, [M+H–H2O]+ and [M+NH4]+ ions, while in ESI− [M–H]-, [M+Cl]−, [M+HCOO]− and [M–H2O]−. CCS values were matched with those predicted by CCSbase platform (https://ccsbase.net/) and CCS-Predict tool by MetaboScape, Smart Formula™ (SF) was used for molecular formula assignement. Manual curation of each lipid was then performed following Lipidomics Standard Initiative (LSI) guidelines (https://lipidomics-standards-initiative.org/guidelines/lipid-species-identification/general-rules), specifically, besides MS/MS diagnostic ions, crucial aspects of lipid annotation such as: (a) lipid adducts in electrospray ionization; (b) regular retention behaviour, e.g., the equivalent carbon number (ECN) model used for RPLC; were carefully evaluated. LipidCreator tool (<https://lifs-tools.org/lipidcreator.html>).

**Proteomics, lipidomics and metabolomics data analysis**

**Data Pre-processing**

Proteomic and metabolomic datasets were normalized by the total ion current, while lipidomic data were normalized using class-specific internal standards. Missing values were imputed with one-fifth of the dataset’s minimum value. Data were subsequently log10-transformed and autoscaled to reduce heteroscedasticity and allow comparison across samples.

**Multivariate Analysis**

Multivariate analysis was performed to explore global trends and age-dependent clustering. Hierarchical clustering and principal component analysis (PCA) were applied to identify overall separation between experimental groups. These approaches allowed the identification of global remodeling of proteomic, metabolomic, and lipidomic profiles associated with aging.

**Univariate Analysis**

Univariate statistical analyses were conducted using N-way ANOVA (p < 0.05) to detect significantly altered features across age groups. Enrichment analyses were then carried out using STRING ([https://string-db.org/](https://string-db.org/?utm_source=chatgpt.com)), ShinyGO 0.82 ([http://bioinformatics.sdstate.edu/go/](http://bioinformatics.sdstate.edu/go/?utm_source=chatgpt.com)), and LipidOne 2.0 ([https://lipidone.eu/](https://lipidone.eu/?utm_source=chatgpt.com)) to identify functional pathways and molecular processes significantly affected by aging.

**Proteomics, metabolomics and lipidomics data processing**

For proteome data processing, raw MS data were analysed using Proteome Discoverer v 2.5 (Thermo Fisher). The analysis was performed with the following parameters: enzyme trypsin, maximum of one missed cleavage, and a mass accuracy tolerance of 10 ppm for precursors and 0.6 Da for fragments The following parameters were used: enzyme trypsin, missed cleavages max 1, mass accuracy tolerance 10 ppm and 0,6 Da for precursors and fragments respectively. Sequest search Percolator and Inferys rescoring algorithm nodes were used. Carbamidomethylcysteine was used as fixed modification while methionine oxidation as variable. Proteins were considered identified with at least one unique peptide, using a false discovery rate (FDR) thresholds of 0.01 (strict) and 1% (relaxed). Each analysis was performed in triplicate.

Metabolomics analysis was performed by Compound Discoverer v.3.3 (Thermo Fisher Scientific) to normalize, align, detect and identify compounds. Features were extracted from 0-10 min and 0-11 min of the HILIC run, respectively, in the m/z=70-800 mass range. Data were aligned according to an adaptive curve alignment model. Compounds were detected using the following parameters settings: mass tolerance was set to 5 ppm, while retention time tolerance was set to 0.2 min; minimum peak intensity was set to 100000 AU and the signal-to-noise threshold for compound detection was set to 5. The peak rating filter was set to 3. To perform blank subtraction, we maintained max sample/max blank > 5. To predict elemental compositions of the compounds, the relative intensity tolerance was set to 30% for isotope pattern matching. For the mzCloud database search, both the precursor and fragment mass tolerance were set to 5 ppm. The databases used for matching compounds in ChemSpider for structural search were BioCyc, the Human Metabolome Database and KEGG, and the mass tolerance in ChemSpider Search was set to 5 ppm. The mass tolerance for matching compounds in Metabolika pathways was set to 5 ppm. Compounds were assigned by comparing annotations using the following nodes in order of priority: (1) mzCloud (2) Predicted Compositions; (3) MassList search; (4) ChemSpider Search; (5) Metabolika search. MetaboScape 2023b (Bruker) was used for lipidomics data processing and lipid annotation. Feature detection was set to 500 and 250 counts for positive and negative modes. The minimum number of data points was set to 100, and recursive feature extraction was used (75 points). Lipid annotation was first performed with a rule-based annotation, based on diagnostic-class specific fragments and their intensity in corresponding MS/MS spectra, and, subsequently, using the LipidBlast spectral library (http://prime.psc.riken.jp/compms/msdial/main.html). The following parameters were set: Mass accuracy window: narrow 2 ppm, wide 10 ppm; mSigma: narrow 30, wide 250, MS/MS score: narrow 800, wide 150. Collision cross-section (CCS) %: narrow 1, wide 3.5. The spectra were processed in ESI+ using as adducts [M+H]+, [M+Na]+, [M+K]+, [M+H–H2O]+ and [M+NH4]+ ions, while in ESI− [M–H]-, [M+Cl]−, [M+HCOO]− and [M–H2O]−. CCS values were matched with those predicted by CCSbase platform (https://ccsbase.net/) and CCS-Predict tool by MetaboScape, Smart Formula™ (SF) was used for molecular formula assignement. Manual curation of each lipid was then performed following Lipidomics Standard Initiative (LSI) guidelines (https://lipidomics-standards-initiative.org/guidelines/lipid-species-identification/general-rules), specifically, besides MS/MS diagnostic ions, crucial aspects of lipid annotation such as: (a) lipid adducts in electrospray ionization; (b) regular retention behaviour, e.g., the equivalent carbon number (ECN) model used for RPLC; were carefully evaluated. LipidCreator tool (https://lifs-tools.org/lipidcreator.html) extension in Skyline (https://skyline.ms/project/home/begin.view) was used for in silico comparison of specific product ions.

Proteomics Enrichment was performed by STRING (Szklarczyk et al. Nucleic Acids Res. 2015 43(Database issue): D447-52) significant proteins ID were uploaded in the multiple protein nodes against Mus Musculus background proteome.

**Supplemental Figure**

**Figure S1:**

**
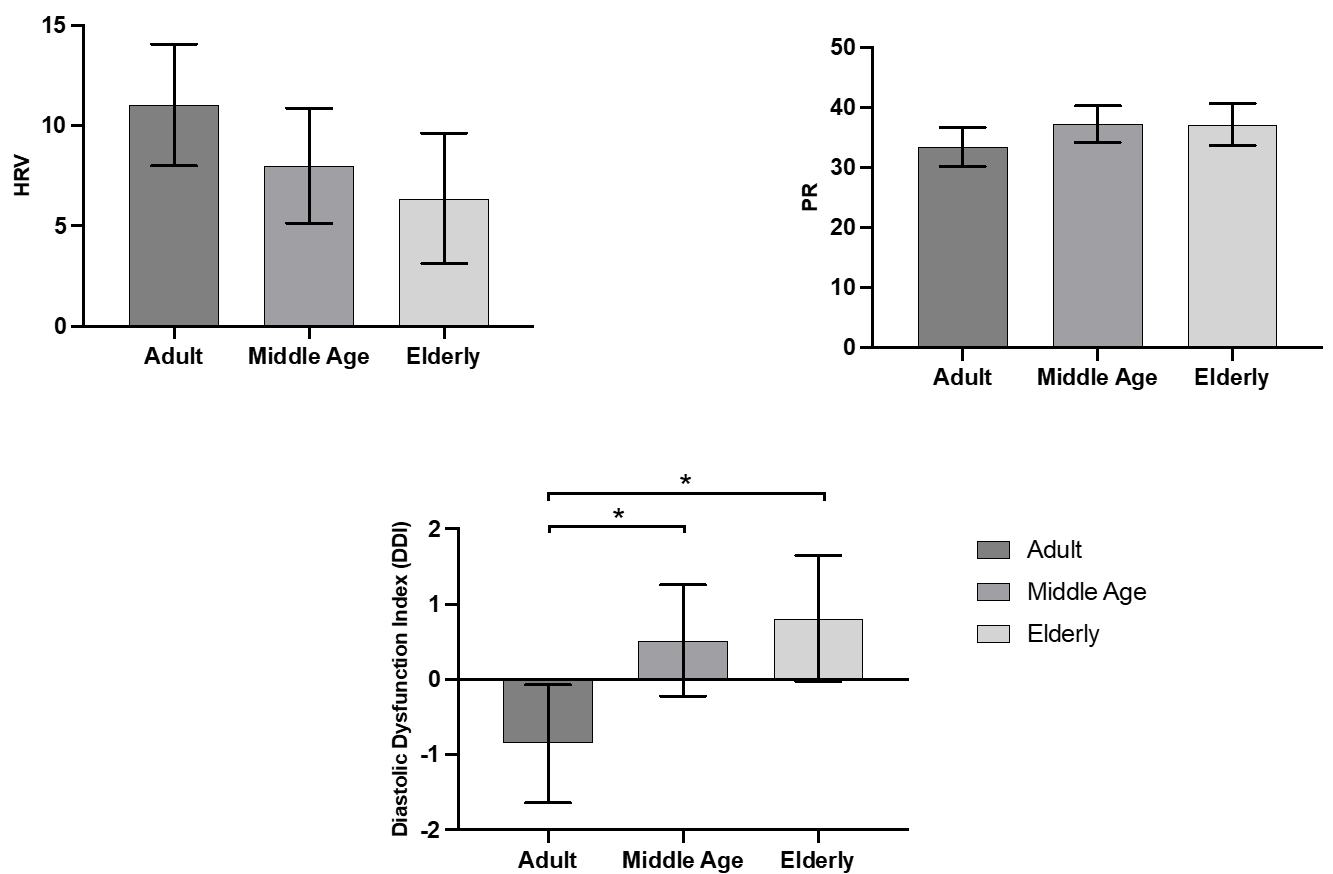
**

**Figure S1:** Electrocardiographic markers of diastolic function (PR interval, HRV) were used to calculate a Diastolic Dysfunction Index (DDI) in mice aged 12, 24, and 30 months. The DDI increased significantly with age, with 30-month-old mice showing the highest values, indicating advanced autonomic and electrical remodeling (one-way ANOVA, p < 0.05). This index distinguished between early and late aging stages.

**Figure S2:**

**
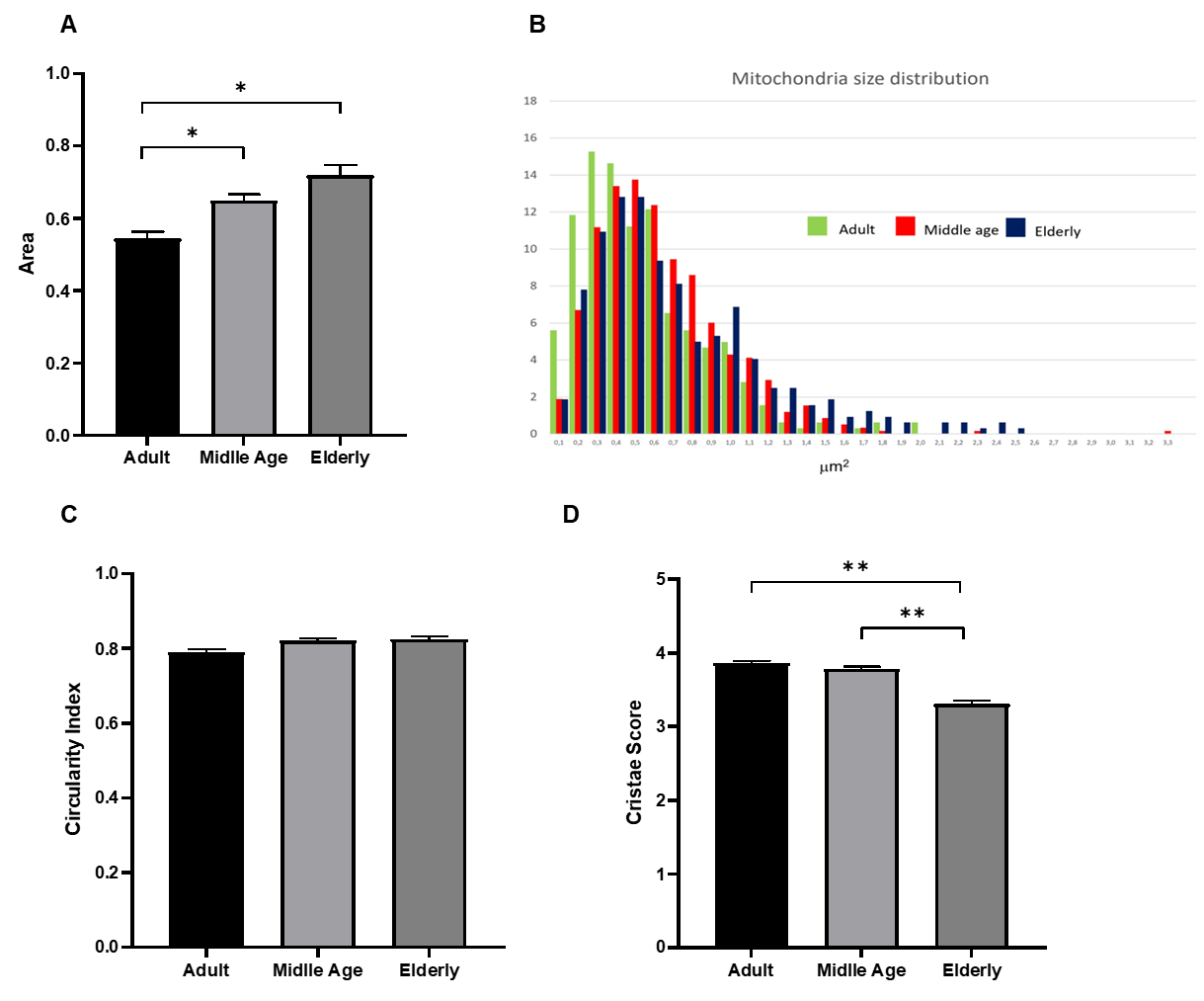
**

**Figure S2:** **A** Bar graph showing a significant increase in mitochondrial Area in the Middle age and Elderly groups compared to the Adult group (*=p<0.05). **B** Histogram of mitochondrial size distribution. The y-axis represents the frequency (number of mitochondria per size bin). The distribution shows a clear shift toward larger mitochondrial areas in the Middle age (red) and Elderly (blue) groups compared to the Adult group (green), indicating a trend toward increased mitochondrial size with aging. **C** Bar graph illustrating mitochondrial Circularity index across the three experimental groups. **D** Bar graph illustrating the mitochondrial Cristae score across the three experimental groups. A significant increase was detected in the elderly group compared with the adult (**p < 0.001) and middle-aged (p* < 0.005) groups.


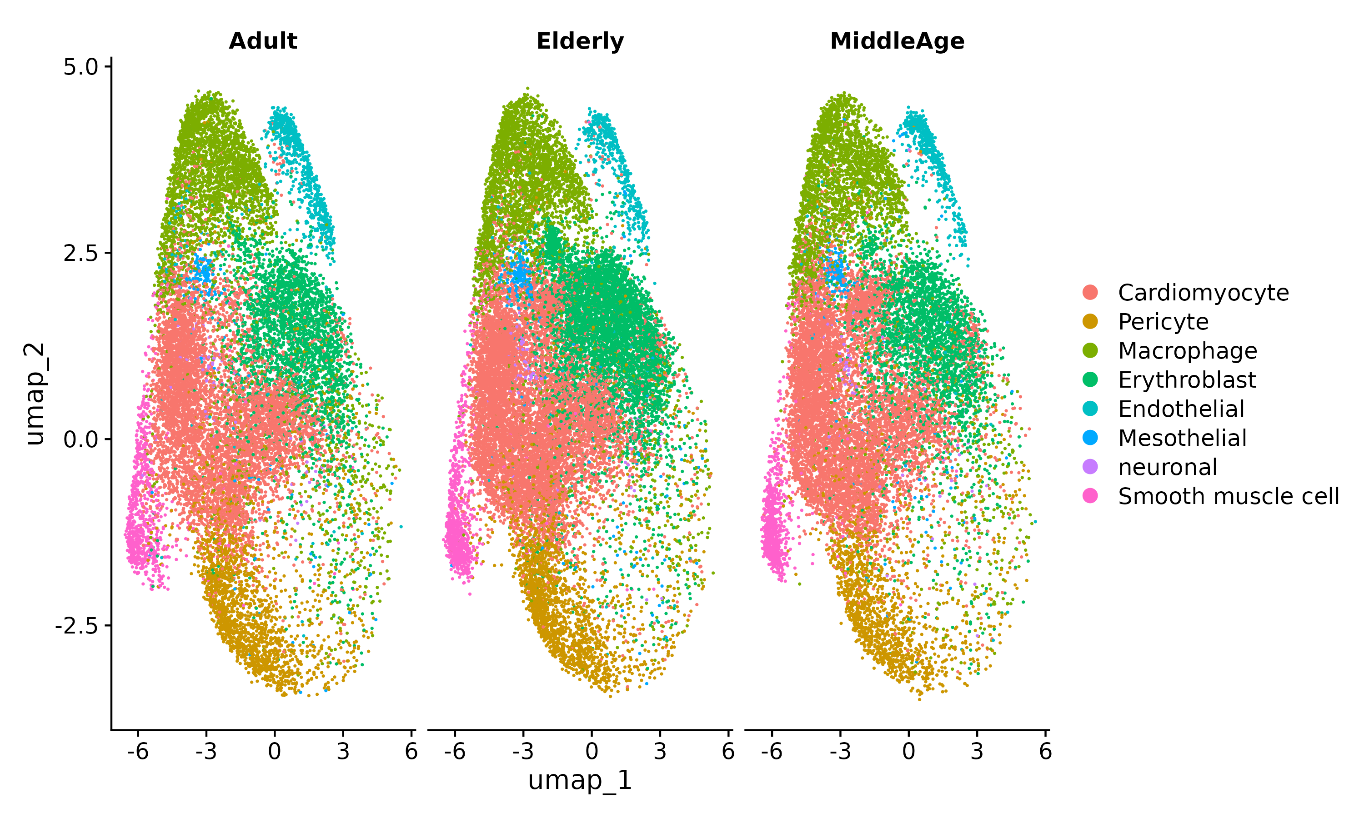


**Figure S3:** UMAP projection of single-cell transcriptomes from adult, middle-aged, and elderly hearts. Each dot represents a single cell, colored according to the annotated cell type. Major cardiac populations are highlighted, including cardiomyocytes, pericytes, macrophages, erythroblasts, endothelial cells, mesothelial cells, neurons, and smooth muscle cells. The distribution across age groups illustrates the preservation of major cell identities with age, while allowing the assessment of subtle shifts in cellular composition and transcriptomic remodeling.


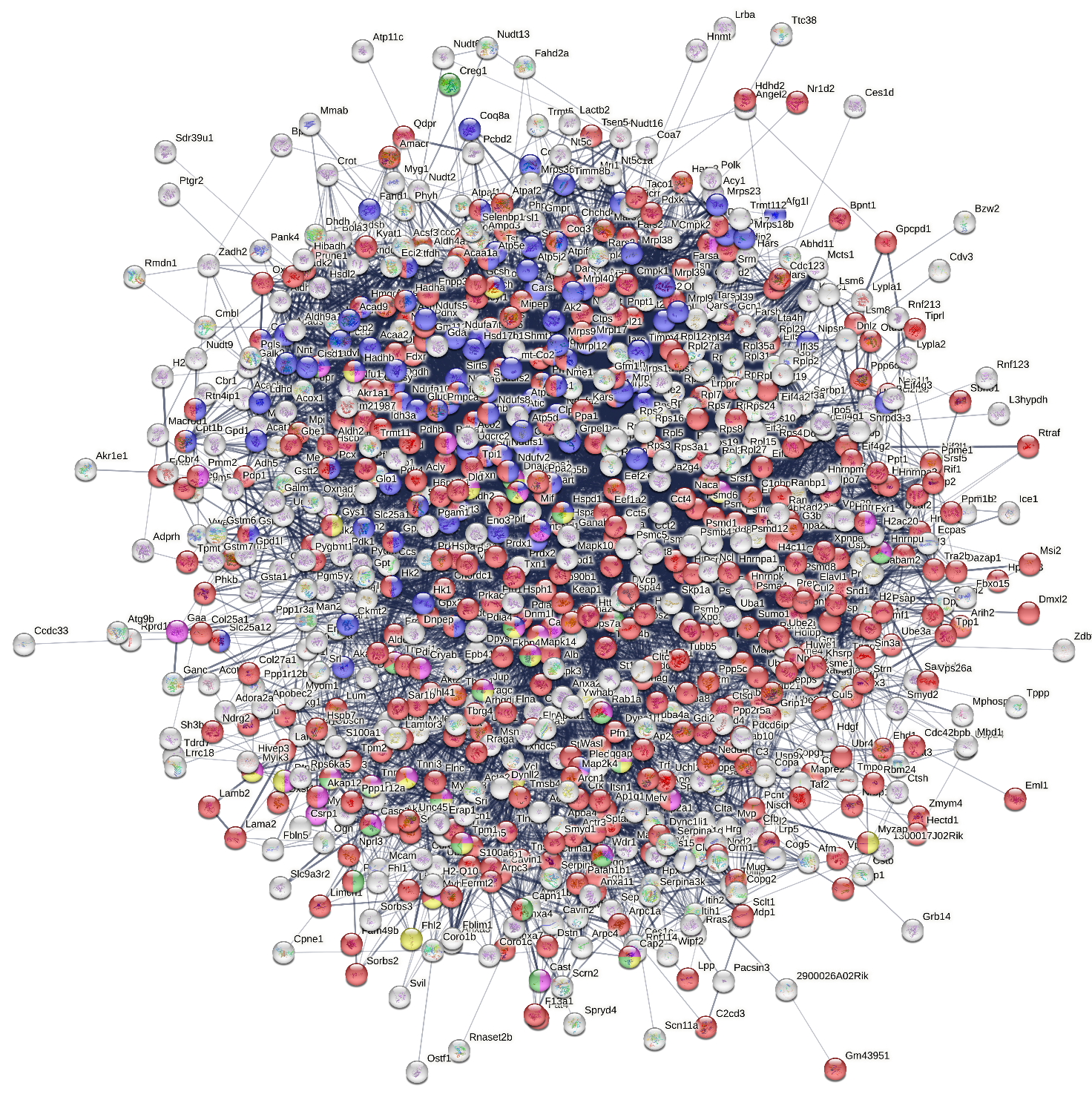


**Figure S4:** String protein-protein network of significant proteins from Anova, colored nodes belong to different enriched pathways, please see figure 3


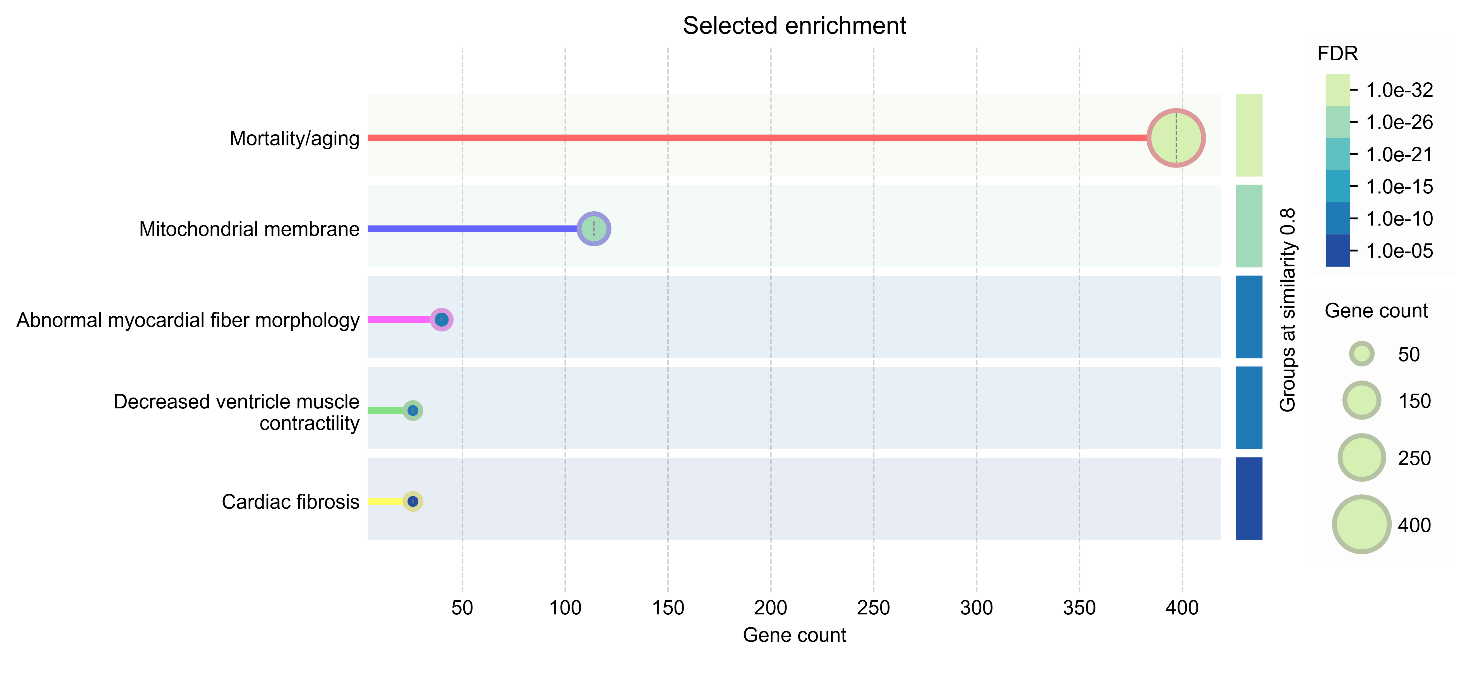


**Figure S5:** Most enriched pathways related to String network shown in figure Figure S4

**Figure S6:** loading plot with explicit metabolite labels shows the distribution of metabolites along the first two loading components (Loadings 1 and Loadings 2). Each point represents a metabolite, with position indicating its relative contribution to the model.


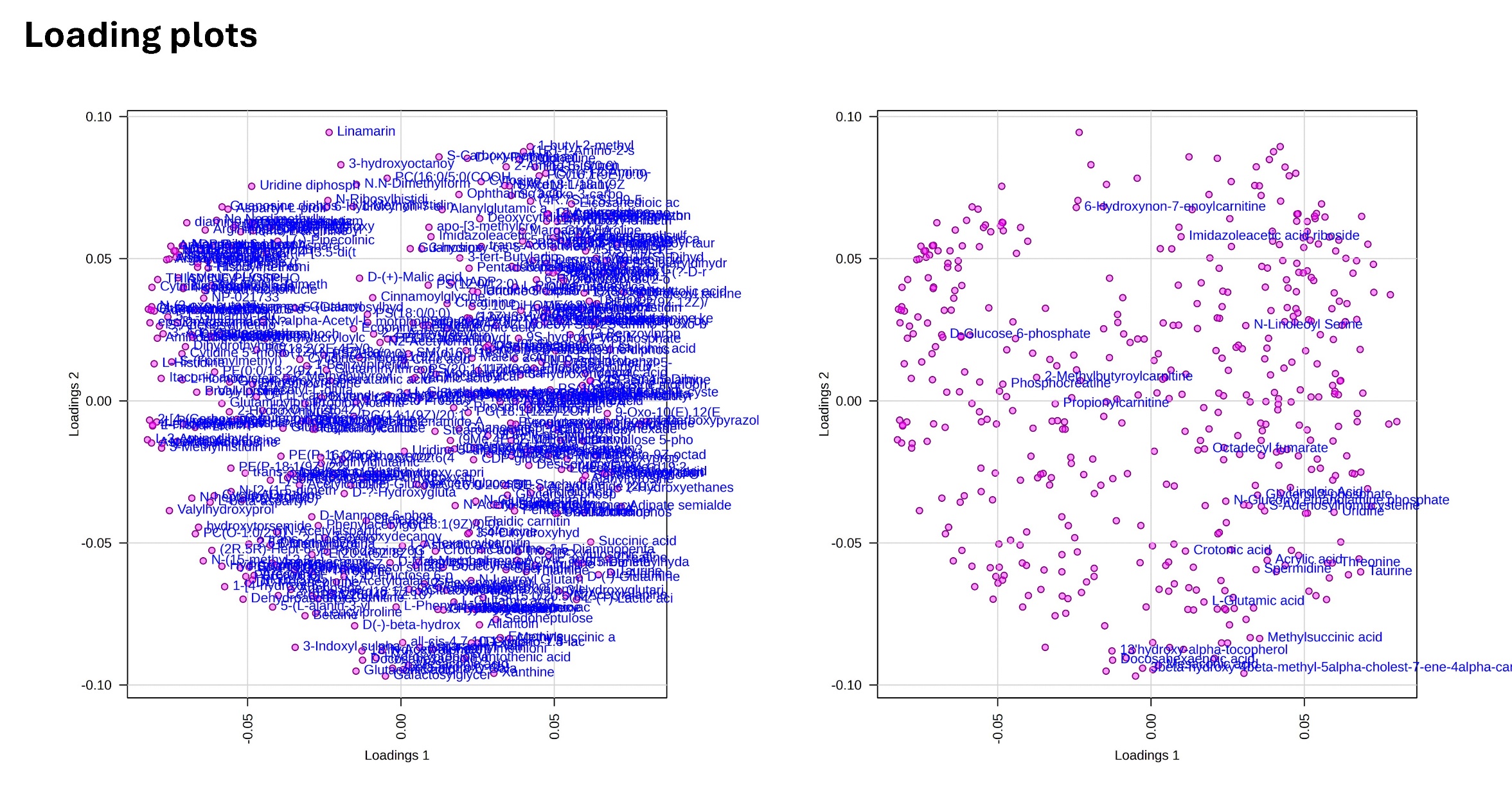


**Table S1:** Table S1: Standard echocardiographic parameters in mice at 6, 12, 24, and 30 months of age, including heart rate (HR), end-diastolic diameter (EDD), end-systolic diameter (ESD), fractional shortening (FS), ejection fraction (EF), and cardiac output (CO).

| **MEAN ± SD** | **HR [beats/min]** | **End-Diastolic Diameter [mm]** | **End-Systolic Diameter [mm]** | **FS [%]** | **EF [%]** | **Cardiac Output [ml/min]** |
| --- | --- | --- | --- | --- | --- | --- |
| **Young** | 458.6 ± 41.28 | 3.033 ± 0.338 | 1.75 ± 0.281 | 42.53 ± 4.362 | 74.93 ± 4.904 | 12.45 ± 2.673 |
| **Adult** | 449.8 ± 54.19 | 3.194 ± 0.367 | 1.861 ± 0.299 | 42.39 ± 5.757 | 74.34 ± 6.464 | 14.26 ± 5.412 |
| **Middle Age** | 418.8 ± 83.6 | 3.387 ± 0.31 | 2.162 ± 0.36 | 35.80 ± 8.96 | 69.80 ± 15.4 | 13.85 ± 4.95 |
| **Elderly** | 424.6 ± 49.0 | 3.112 ± 0.45 | 1.962 ± 0.40 | 37.16 ± 7.04 | 67.81 ± 8.41 | 11.31 ± 4.28 |

**Table S2-S4:** Anova results from proteomics (S2), metabolomics (S3) and lipidomics (S4) reporting Unprot ID and metabolite-lipid annotation, respectively.

| **UniprotID** | **p.value** | **FDR** |
| --- | --- | --- |
| Q9JM14 | 6.03E-17 | 7.51E-14 |
| P09813 | 1.19E-16 | 7.51E-14 |
| Q3U0V1 | 2.00E-16 | 7.51E-14 |
| P13634 | 2.05E-16 | 7.51E-14 |
| Q91VF2 | 3.26E-16 | 9.57E-14 |
| P62849 | 1.03E-15 | 2.24E-13 |
| Q9DBD0 | 1.16E-15 | 2.24E-13 |
| O09061 | 1.26E-15 | 2.24E-13 |
| Q8BTM8 | 1.38E-15 | 2.24E-13 |
| P52196 | 2.87E-15 | 4.21E-13 |
| P06330 | 3.99E-15 | 5.32E-13 |
| Q9WVH9 | 5.20E-15 | 6.35E-13 |
| Q6P9R2 | 7.91E-15 | 8.92E-13 |
| Q80X85 | 1.41E-14 | 1.48E-12 |
| Q62407 | 8.17E-14 | 7.98E-12 |
| Q60668 | 2.42E-13 | 2.21E-11 |
| P17426 | 3.03E-13 | 2.62E-11 |
| P42567 | 4.29E-13 | 3.26E-11 |
| Q99JW2 | 4.42E-13 | 3.26E-11 |
| P28656 | 4.61E-13 | 3.26E-11 |
| Q8K1M6 | 4.67E-13 | 3.26E-11 |
| Q8BGD8 | 4.98E-13 | 3.32E-11 |
| P63166 | 1.46E-12 | 9.33E-11 |
| P70227 | 2.44E-12 | 1.49E-10 |
| Q9JJZ2 | 2.55E-12 | 1.49E-10 |
| P13745 | 2.84E-12 | 1.60E-10 |
| Q8R001 | 6.07E-12 | 3.30E-10 |
| Q9DCH4 | 6.31E-12 | 3.30E-10 |
| Q9Z1P6 | 6.76E-12 | 3.42E-10 |
| O35887 | 1.41E-11 | 6.87E-10 |
| Q91W90 | 1.86E-11 | 8.78E-10 |
| P28665 | 3.23E-11 | 1.44E-09 |
| Q9QY76 | 3.27E-11 | 1.44E-09 |
| O70325 | 3.33E-11 | 1.44E-09 |
| E9PVA8 | 4.88E-11 | 2.04E-09 |
| Q9QZN0 | 5.30E-11 | 2.11E-09 |
| Q9EQH2 | 5.42E-11 | 2.11E-09 |
| P49138 | 5.48E-11 | 2.11E-09 |
| Q9CZL5 | 8.38E-11 | 3.15E-09 |
| Q8R104 | 1.06E-10 | 3.87E-09 |
| Q61035 | 1.45E-10 | 5.18E-09 |
| O55131 | 1.82E-10 | 6.37E-09 |
| Q60866 | 2.12E-10 | 7.24E-09 |
| Q64433 | 2.86E-10 | 9.53E-09 |
| Q9EQK5 | 4.36E-10 | 1.41E-08 |
| P28798 | 4.41E-10 | 1.41E-08 |
| Q9QXK3 | 6.14E-10 | 1.91E-08 |
| P54728 | 7.22E-10 | 2.19E-08 |
| P61982 | 7.31E-10 | 2.19E-08 |
| Q9WTX5 | 8.17E-10 | 2.40E-08 |
| Q9CQE3 | 9.12E-10 | 2.62E-08 |
| O08739 | 9.52E-10 | 2.65E-08 |
| Q9WTR5 | 9.60E-10 | 2.65E-08 |
| P26883 | 9.75E-10 | 2.65E-08 |
| P47964 | 1.00E-09 | 2.66E-08 |
| Q8BHL8 | 1.17E-09 | 3.06E-08 |
| P97315 | 1.72E-09 | 4.42E-08 |
| Q9QZE5 | 1.88E-09 | 4.76E-08 |
| Q9D6R2 | 2.06E-09 | 5.11E-08 |
| Q9CZR8 | 2.09E-09 | 5.11E-08 |
| O08677 | 2.25E-09 | 5.42E-08 |
| Q9ET26 | 2.29E-09 | 5.42E-08 |
| Q8BHN3 | 2.42E-09 | 5.62E-08 |
| P97384 | 2.67E-09 | 6.12E-08 |
| Q02257 | 2.86E-09 | 6.45E-08 |
| Q61941 | 3.06E-09 | 6.79E-08 |
| Q7TT50 | 3.15E-09 | 6.90E-08 |
| Q8CDI6 | 3.42E-09 | 7.37E-08 |
| Q6P3D0 | 3.54E-09 | 7.53E-08 |
| Q60590 | 4.30E-09 | 9.00E-08 |
| P55302 | 4.40E-09 | 9.08E-08 |
| P47738 | 4.50E-09 | 9.17E-08 |
| P62889 | 4.72E-09 | 9.48E-08 |
| B0F2B4 | 4.84E-09 | 9.60E-08 |
| P05201 | 5.65E-09 | 1.10E-07 |
| Q99J45 | 6.22E-09 | 1.20E-07 |
| Q9WVJ2 | 6.63E-09 | 1.26E-07 |
| P53996 | 7.32E-09 | 1.38E-07 |
| Q9WUM3 | 7.88E-09 | 1.46E-07 |
| Q9CQE8 | 8.21E-09 | 1.50E-07 |
| Q99KR3 | 8.36E-09 | 1.51E-07 |
| P54775 | 8.44E-09 | 1.51E-07 |
| Q9CZ04 | 8.69E-09 | 1.54E-07 |
| P17918 | 9.87E-09 | 1.72E-07 |
| P97371 | 1.06E-08 | 1.83E-07 |
| Q91YM4 | 1.09E-08 | 1.87E-07 |
| P26443 | 1.26E-08 | 2.13E-07 |
| P36993 | 1.30E-08 | 2.17E-07 |
| Q9D8C4 | 1.35E-08 | 2.22E-07 |
| Q8R1Q8 | 1.41E-08 | 2.29E-07 |
| Q8K1C0 | 1.42E-08 | 2.29E-07 |
| P29595 | 1.45E-08 | 2.31E-07 |
| Q4VAA2 | 1.46E-08 | 2.31E-07 |
| Q8CIG8 | 1.53E-08 | 2.38E-07 |
| Q8BG05 | 1.72E-08 | 2.63E-07 |
| Q69ZR2 | 1.72E-08 | 2.63E-07 |
| Q9CSU0 | 1.74E-08 | 2.63E-07 |
| Q9D7N3 | 1.80E-08 | 2.69E-07 |
| Q9CPP6 | 2.02E-08 | 3.00E-07 |
| Q9CQZ5 | 2.09E-08 | 3.05E-07 |
| Q9JK81 | 2.10E-08 | 3.05E-07 |
| P97765 | 2.31E-08 | 3.29E-07 |
| Q99N94 | 2.32E-08 | 3.29E-07 |
| O89079 | 2.33E-08 | 3.29E-07 |
| Q91X72 | 2.36E-08 | 3.29E-07 |
| Q63918 | 2.51E-08 | 3.44E-07 |
| Q99KK9 | 2.51E-08 | 3.44E-07 |
| Q9JJU8 | 2.65E-08 | 3.58E-07 |
| Q80XI3 | 2.66E-08 | 3.58E-07 |
| Q9WUA2 | 2.73E-08 | 3.63E-07 |
| Q8BH61 | 2.85E-08 | 3.75E-07 |
| Q80TM9 | 2.88E-08 | 3.75E-07 |
| Q9DCS2 | 2.90E-08 | 3.75E-07 |
| Q8K3J1 | 2.92E-08 | 3.75E-07 |
| Q3UH68 | 3.13E-08 | 3.99E-07 |
| Q8R5H1 | 3.37E-08 | 4.26E-07 |
| P99027 | 3.63E-08 | 4.55E-07 |
| Q61990 | 4.29E-08 | 5.33E-07 |
| Q9D8W5 | 4.69E-08 | 5.78E-07 |
| Q9ESB3 | 4.76E-08 | 5.79E-07 |
| Q5SW19 | 4.78E-08 | 5.79E-07 |
| Q3UV70 | 5.01E-08 | 6.03E-07 |
| Q8CII2 | 5.26E-08 | 6.27E-07 |
| Q9CQX8 | 6.71E-08 | 7.93E-07 |
| O54724 | 6.77E-08 | 7.94E-07 |
| O08715 | 6.92E-08 | 8.05E-07 |
| Q8R5A0 | 7.43E-08 | 8.58E-07 |
| Q99M01 | 7.53E-08 | 8.63E-07 |
| Q99LY9 | 7.73E-08 | 8.78E-07 |
| Q7TSH2 | 8.91E-08 | 1.00E-06 |
| Q6PR54 | 9.81E-08 | 1.10E-06 |
| Q9Z1K6 | 1.01E-07 | 1.12E-06 |
| Q99MQ5 | 1.08E-07 | 1.19E-06 |
| Q80X90 | 1.14E-07 | 1.25E-06 |
| P62918 | 1.20E-07 | 1.30E-06 |
| Q8BFY9 | 1.25E-07 | 1.34E-06 |
| P70168 | 1.48E-07 | 1.58E-06 |
| Q9Z0P5 | 1.77E-07 | 1.88E-06 |
| P06909 | 2.02E-07 | 2.13E-06 |
| P62855 | 2.04E-07 | 2.13E-06 |
| P14131 | 2.31E-07 | 2.41E-06 |
| O88668 | 2.66E-07 | 2.75E-06 |
| P01864 | 3.00E-07 | 3.08E-06 |
| Q62234 | 3.30E-07 | 3.36E-06 |
| Q925T6 | 3.81E-07 | 3.85E-06 |
| Q60613 | 3.94E-07 | 3.95E-06 |
| Q7TPR4 | 4.14E-07 | 4.13E-06 |
| P50544 | 4.23E-07 | 4.19E-06 |
| Q61879 | 4.48E-07 | 4.41E-06 |
| Q9CYT6 | 4.62E-07 | 4.51E-06 |
| P62082 | 4.69E-07 | 4.55E-06 |
| Q9CQ80 | 4.78E-07 | 4.61E-06 |
| Q9JHU4 | 5.26E-07 | 5.04E-06 |
| P47199 | 5.55E-07 | 5.28E-06 |
| Q3TXS7 | 5.59E-07 | 5.29E-06 |
| P49312 | 5.77E-07 | 5.42E-06 |
| Q64523 | 6.70E-07 | 6.25E-06 |
| Q8BH58 | 6.83E-07 | 6.34E-06 |
| Q9CZN8 | 6.92E-07 | 6.38E-06 |
| Q61545 | 7.09E-07 | 6.50E-06 |
| Q9D1R9 | 7.14E-07 | 6.50E-06 |
| O08600 | 8.05E-07 | 7.25E-06 |
| Q8BP40 | 8.13E-07 | 7.25E-06 |
| Q9D6J6 | 8.13E-07 | 7.25E-06 |
| P70195 | 8.16E-07 | 7.25E-06 |
| P68037 | 8.75E-07 | 7.70E-06 |
| Q9CXT8 | 8.77E-07 | 7.70E-06 |
| Q61584 | 8.96E-07 | 7.82E-06 |
| P61089 | 9.10E-07 | 7.90E-06 |
| Q8VEK3 | 9.37E-07 | 8.08E-06 |
| Q6PH08 | 9.67E-07 | 8.29E-06 |
| P50462 | 9.90E-07 | 8.44E-06 |
| Q68FD5 | 1.01E-06 | 8.53E-06 |
| Q7TNG5 | 1.03E-06 | 8.71E-06 |
| O35857 | 1.04E-06 | 8.73E-06 |
| Q9D8P4 | 1.05E-06 | 8.77E-06 |
| P61979 | 1.09E-06 | 9.06E-06 |
| P10630 | 1.15E-06 | 9.47E-06 |
| Q6P2L7 | 1.17E-06 | 9.53E-06 |
| Q6P069 | 1.17E-06 | 9.53E-06 |
| Q99N87 | 1.24E-06 | 1.01E-05 |
| Q60597 | 1.31E-06 | 1.06E-05 |
| P53026 | 1.34E-06 | 1.07E-05 |
| P50752 | 1.37E-06 | 1.09E-05 |
| Q9D2N4 | 1.50E-06 | 1.19E-05 |
| Q921H8 | 1.53E-06 | 1.20E-05 |
| P80315 | 1.53E-06 | 1.20E-05 |
| Q922R8 | 1.80E-06 | 1.41E-05 |
| Q6PDI5 | 1.85E-06 | 1.43E-05 |
| P07759 | 1.86E-06 | 1.43E-05 |
| P70372 | 1.86E-06 | 1.43E-05 |
| Q7TMY8 | 1.94E-06 | 1.48E-05 |
| Q99N84 | 1.95E-06 | 1.48E-05 |
| Q9D1Q6 | 1.96E-06 | 1.48E-05 |
| Q9DC61 | 2.07E-06 | 1.56E-05 |
| Q6ZWN5 | 2.11E-06 | 1.58E-05 |
| Q61147 | 2.13E-06 | 1.58E-05 |
| Q8VCM7 | 2.18E-06 | 1.61E-05 |
| P62702 | 2.19E-06 | 1.61E-05 |
| Q8BVU5 | 2.19E-06 | 1.61E-05 |
| Q9JKF7 | 2.23E-06 | 1.62E-05 |
| P49813 | 2.44E-06 | 1.77E-05 |
| Q8BU30 | 2.51E-06 | 1.81E-05 |
| O35350 | 2.52E-06 | 1.81E-05 |
| Q3TEA8 | 2.63E-06 | 1.88E-05 |
| Q3TCH7 | 2.65E-06 | 1.88E-05 |
| Q91WS0 | 2.66E-06 | 1.89E-05 |
| P47911 | 2.72E-06 | 1.91E-05 |
| Q7TQ48 | 2.75E-06 | 1.93E-05 |
| Q9Z1E4 | 2.86E-06 | 1.99E-05 |
| Q00519 | 3.06E-06 | 2.12E-05 |
| Q9WV92 | 3.18E-06 | 2.19E-05 |
| P42859 | 3.19E-06 | 2.19E-05 |
| Q9ESE1 | 3.23E-06 | 2.21E-05 |
| P61082 | 3.29E-06 | 2.24E-05 |
| P35385 | 3.45E-06 | 2.34E-05 |
| Q99KQ4 | 3.60E-06 | 2.43E-05 |
| Q9CX56 | 3.61E-06 | 2.43E-05 |
| Q9R112 | 3.69E-06 | 2.46E-05 |
| P25444 | 3.70E-06 | 2.46E-05 |
| P46471 | 3.78E-06 | 2.50E-05 |
| Q9Z2Q5 | 3.78E-06 | 2.50E-05 |
| P02468 | 3.84E-06 | 2.52E-05 |
| Q921M7 | 3.89E-06 | 2.54E-05 |
| P35700 | 4.03E-06 | 2.62E-05 |
| Q9QUR6 | 4.13E-06 | 2.67E-05 |
| Q3TMH2 | 4.14E-06 | 2.67E-05 |
| Q6P1B1 | 4.29E-06 | 2.76E-05 |
| Q9Z1Q9 | 4.59E-06 | 2.94E-05 |
| P48722 | 4.70E-06 | 3.00E-05 |
| A2AN08 | 4.90E-06 | 3.11E-05 |
| Q6P5E4 | 4.99E-06 | 3.15E-05 |
| Q3UHB1 | 5.10E-06 | 3.21E-05 |
| Q9DCX2 | 5.23E-06 | 3.27E-05 |
| Q60649 | 5.25E-06 | 3.28E-05 |
| Q9DB15 | 5.79E-06 | 3.60E-05 |
| Q9QZ88 | 6.12E-06 | 3.78E-05 |
| P35979 | 6.23E-06 | 3.84E-05 |
| Q8QZR5 | 6.28E-06 | 3.85E-05 |
| Q80VM7 | 6.38E-06 | 3.89E-05 |
| Q8CH40 | 6.52E-06 | 3.96E-05 |
| O70318 | 6.62E-06 | 4.01E-05 |
| Q61578 | 6.99E-06 | 4.22E-05 |
| Q99JY9 | 7.15E-06 | 4.29E-05 |
| Q8K4L3 | 7.19E-06 | 4.30E-05 |
| Q9JM76 | 7.96E-06 | 4.74E-05 |
| P06728 | 8.32E-06 | 4.94E-05 |
| Q9Z2C5 | 8.69E-06 | 5.14E-05 |
| P63005 | 9.07E-06 | 5.34E-05 |
| Q5SSW2 | 9.21E-06 | 5.40E-05 |
| Q8VDM4 | 9.47E-06 | 5.53E-05 |
| A6H611 | 9.53E-06 | 5.55E-05 |
| Q8K0Z7 | 1.00E-05 | 5.81E-05 |
| C0HKG6 | 1.02E-05 | 5.89E-05 |
| P46412 | 1.04E-05 | 5.93E-05 |
| Q9WVK4 | 1.04E-05 | 5.93E-05 |
| Q62188 | 1.06E-05 | 5.99E-05 |
| Q3UIJ9 | 1.06E-05 | 5.99E-05 |
| Q9QZW0 | 1.07E-05 | 5.99E-05 |
| Q9CQ07 | 1.07E-05 | 5.99E-05 |
| A2AAJ9 | 1.07E-05 | 5.99E-05 |
| Q8C0L9 | 1.11E-05 | 6.22E-05 |
| Q920Q6 | 1.13E-05 | 6.30E-05 |
| O70250 | 1.17E-05 | 6.51E-05 |
| P20065 | 1.22E-05 | 6.76E-05 |
| Q8K2H2 | 1.23E-05 | 6.79E-05 |
| Q9EQ80 | 1.24E-05 | 6.81E-05 |
| Q6DYE8 | 1.28E-05 | 6.98E-05 |
| Q9CQR6 | 1.28E-05 | 6.98E-05 |
| P35282 | 1.31E-05 | 7.12E-05 |
| Q9CPR5 | 1.37E-05 | 7.36E-05 |
| P97429 | 1.37E-05 | 7.36E-05 |
| Q9R111 | 1.37E-05 | 7.36E-05 |
| Q61553 | 1.38E-05 | 7.36E-05 |
| P50171 | 1.42E-05 | 7.59E-05 |
| P12367 | 1.48E-05 | 7.85E-05 |
| Q9QXS1 | 1.50E-05 | 7.91E-05 |
| Q9QZ06 | 1.51E-05 | 7.95E-05 |
| Q9R1P3 | 1.52E-05 | 7.99E-05 |
| E9Q4Z2 | 1.53E-05 | 7.99E-05 |
| P58252 | 1.53E-05 | 7.99E-05 |
| Q99JB8 | 1.54E-05 | 8.01E-05 |
| P14115 | 1.55E-05 | 8.02E-05 |
| Q8VE22 | 1.63E-05 | 8.42E-05 |
| P80314 | 1.64E-05 | 8.42E-05 |
| P09405 | 1.65E-05 | 8.47E-05 |
| Q6EBV9 | 1.66E-05 | 8.48E-05 |
| Q60675 | 1.67E-05 | 8.49E-05 |
| Q9R1P4 | 1.69E-05 | 8.58E-05 |
| P62301 | 1.72E-05 | 8.68E-05 |
| P61202 | 1.73E-05 | 8.70E-05 |
| P97351 | 1.77E-05 | 8.87E-05 |
| Q9CXA2 | 1.83E-05 | 9.14E-05 |
| Q3U186 | 1.85E-05 | 9.24E-05 |
| Q8C0L8 | 1.86E-05 | 9.25E-05 |
| Q8BKC5 | 1.89E-05 | 9.36E-05 |
| Q8BKY8 | 1.96E-05 | 9.66E-05 |
| Q9DCT1 | 1.99E-05 | 9.80E-05 |
| Q9DBJ1 | 2.01E-05 | 9.86E-05 |
| P62320 | 2.02E-05 | 9.88E-05 |
| Q11011 | 2.06E-05 | 0.0001 |
| P08030 | 2.10E-05 | 0.000102 |
| Q60676 | 2.11E-05 | 0.000102 |
| Q924D0 | 2.11E-05 | 0.000102 |
| Q8K2Y7 | 2.20E-05 | 0.000106 |
| Q3ULW6 | 2.21E-05 | 0.000106 |
| Q61702 | 2.21E-05 | 0.000106 |
| P61164 | 2.25E-05 | 0.000107 |
| Q8VHX6 | 2.25E-05 | 0.000107 |
| Q91YP0 | 2.28E-05 | 0.000108 |
| Q9CXJ1 | 2.30E-05 | 0.000108 |
| Q9CR57 | 2.36E-05 | 0.000111 |
| Q8CC88 | 2.36E-05 | 0.000111 |
| P23198 | 2.37E-05 | 0.000111 |
| Q3TYA6 | 2.44E-05 | 0.000114 |
| Q921H9 | 2.45E-05 | 0.000114 |
| Q02053 | 2.49E-05 | 0.000115 |
| Q6NZJ6 | 2.51E-05 | 0.000116 |
| Q8BGC4 | 2.58E-05 | 0.000119 |
| P26043 | 2.59E-05 | 0.000119 |
| Q9D967 | 2.60E-05 | 0.000119 |
| P40336 | 2.71E-05 | 0.000123 |
| O55234 | 2.73E-05 | 0.000124 |
| Q91VR2 | 2.94E-05 | 0.000133 |
| A2A884 | 3.09E-05 | 0.000139 |
| P23927 | 3.13E-05 | 0.000141 |
| Q8BTY1 | 3.14E-05 | 0.000141 |
| A3KMP2 | 3.16E-05 | 0.000141 |
| P26369 | 3.20E-05 | 0.000143 |
| Q9D0C4 | 3.22E-05 | 0.000143 |
| Q9QYG0 | 3.43E-05 | 0.000152 |
| Q9DB77 | 3.50E-05 | 0.000154 |
| Q9Z2E2 | 3.67E-05 | 0.000162 |
| P97443 | 3.73E-05 | 0.000164 |
| Q9D113 | 3.76E-05 | 0.000165 |
| P17710 | 3.87E-05 | 0.000169 |
| Q8QZY1 | 3.88E-05 | 0.000169 |
| P31750 | 3.89E-05 | 0.000169 |
| P50580 | 3.95E-05 | 0.000171 |
| Q9WU78 | 4.15E-05 | 0.000179 |
| Q9CQ60 | 4.27E-05 | 0.000183 |
| Q61838 | 4.35E-05 | 0.000186 |
| Q61831 | 4.44E-05 | 0.00019 |
| P06801 | 4.55E-05 | 0.000194 |
| P03921 | 4.74E-05 | 0.000201 |
| Q9CXI0 | 4.82E-05 | 0.000204 |
| Q9JK42 | 4.84E-05 | 0.000204 |
| Q3UTJ2 | 5.02E-05 | 0.000211 |
| O88712 | 5.18E-05 | 0.000217 |
| Q9D3D9 | 5.36E-05 | 0.000225 |
| Q8BH64 | 5.39E-05 | 0.000225 |
| O88952 | 5.56E-05 | 0.000232 |
| P12970 | 5.59E-05 | 0.000232 |
| Q04447 | 5.71E-05 | 0.000236 |
| Q8VEA4 | 5.78E-05 | 0.000239 |
| A2AUC9 | 5.99E-05 | 0.000247 |
| Q91V92 | 6.04E-05 | 0.000248 |
| Q01853 | 6.07E-05 | 0.000249 |
| P13707 | 6.10E-05 | 0.000249 |
| Q8JZN5 | 6.24E-05 | 0.000254 |
| P01027 | 6.33E-05 | 0.000257 |
| Q91VT4 | 6.62E-05 | 0.000268 |
| Q9DCM2 | 6.65E-05 | 0.000269 |
| Q6PD03 | 6.78E-05 | 0.000273 |
| Q8C2A2 | 7.64E-05 | 0.000307 |
| P62908 | 7.98E-05 | 0.000319 |
| Q9JMA1 | 8.02E-05 | 0.00032 |
| Q8BZF8 | 8.08E-05 | 0.000322 |
| Q9CQC9 | 8.49E-05 | 0.000337 |
| P23953 | 8.64E-05 | 0.000343 |
| Q9R053 | 8.89E-05 | 0.000351 |
| Q60520 | 9.02E-05 | 0.000355 |
| Q5QNQ9 | 9.07E-05 | 0.000356 |
| Q9DBB8 | 9.21E-05 | 0.000361 |
| P61922 | 9.37E-05 | 0.000366 |
| Q9CZS1 | 9.87E-05 | 0.000385 |
| P62245 | 0.000102 | 0.000396 |
| Q9WV32 | 0.000102 | 0.000396 |
| Q9CWE0 | 0.000103 | 0.000397 |
| P01867 | 0.000103 | 0.000397 |
| Q9WTQ5 | 0.000104 | 0.0004 |
| E9Q555 | 0.000105 | 0.000402 |
| Q8BGD9 | 0.000106 | 0.000406 |
| P97447 | 0.000109 | 0.000414 |
| Q91YD9 | 0.000109 | 0.000415 |
| P97450 | 0.00011 | 0.000419 |
| Q9CQW1 | 0.000113 | 0.000427 |
| Q8BML9 | 0.000113 | 0.000427 |
| Q9D5V5 | 0.000113 | 0.000427 |
| P27773 | 0.000114 | 0.00043 |
| Q9JJH7 | 0.000115 | 0.00043 |
| P62962 | 0.000117 | 0.000435 |
| Q64727 | 0.000117 | 0.000435 |
| P0CC03 | 0.00012 | 0.000447 |
| P53395 | 0.000121 | 0.000448 |
| P97855 | 0.000122 | 0.000453 |
| Q68FH4 | 0.000123 | 0.000453 |
| P30416 | 0.000124 | 0.000457 |
| P56135 | 0.000125 | 0.000458 |
| Q61234 | 0.000128 | 0.000469 |
| Q61171 | 0.000128 | 0.000469 |
| Q64674 | 0.000136 | 0.000496 |
| Q8K2C6 | 0.000138 | 0.000504 |
| Q05BC3 | 0.000139 | 0.000505 |
| Q91W89 | 0.000151 | 0.000545 |
| Q8BH59 | 0.000155 | 0.000558 |
| Q6ZQ73 | 0.000155 | 0.000558 |
| Q9CPU0 | 0.000155 | 0.000558 |
| P59017 | 0.000156 | 0.000559 |
| Q9D8N0 | 0.000158 | 0.000564 |
| Q2TPA8 | 0.00016 | 0.00057 |
| P60670 | 0.000161 | 0.000571 |
| O88342 | 0.000162 | 0.000574 |
| Q9JIY5 | 0.000167 | 0.000593 |
| O70571 | 0.000168 | 0.000593 |
| O35326 | 0.000169 | 0.000597 |
| Q9D1P4 | 0.00017 | 0.000598 |
| P01878 | 0.000171 | 0.000599 |
| Q9CZM2 | 0.000172 | 0.0006 |
| Q9D6Y9 | 0.000173 | 0.000605 |
| Q921S7 | 0.000177 | 0.000615 |
| P99026 | 0.000177 | 0.000615 |
| P54923 | 0.000181 | 0.000629 |
| Q01768 | 0.000183 | 0.000633 |
| Q9DAK9 | 0.000187 | 0.000645 |
| P14069 | 0.000197 | 0.000679 |
| Q99MN1 | 0.000204 | 0.000699 |
| Q8BYM8 | 0.00021 | 0.00072 |
| P29341 | 0.000214 | 0.000732 |
| Q8C1A5 | 0.000216 | 0.000736 |
| Q99NB1 | 0.000216 | 0.000736 |
| O54984 | 0.000218 | 0.000741 |
| P47809 | 0.00022 | 0.000744 |
| P14148 | 0.000222 | 0.000749 |
| Q3THS6 | 0.000222 | 0.000749 |
| O88569 | 0.000225 | 0.000757 |
| Q9DBG3 | 0.000234 | 0.000786 |
| Q8BVG4 | 0.000241 | 0.000805 |
| O88587 | 0.000244 | 0.000816 |
| P63101 | 0.000253 | 0.000842 |
| P50431 | 0.000254 | 0.000843 |
| Q9Z2I8 | 0.000254 | 0.000843 |
| Q9D819 | 0.000258 | 0.000855 |
| Q9WV35 | 0.000259 | 0.000855 |
| Q8BMS4 | 0.000266 | 0.000877 |
| P59325 | 0.000273 | 0.000898 |
| Q9JII6 | 0.000274 | 0.000899 |
| O08749 | 0.000275 | 0.000899 |
| Q6P5F9 | 0.000276 | 0.0009 |
| Q3TC72 | 0.000278 | 0.000905 |
| E9Q286 | 0.000281 | 0.000912 |
| Q63844 | 0.000281 | 0.000912 |
| Q9WTP7 | 0.000282 | 0.000912 |
| Q61166 | 0.000284 | 0.000917 |
| Q60710 | 0.000288 | 0.000927 |
| Q99JW4 | 0.000293 | 0.000943 |
| Q9JHK4 | 0.0003 | 0.000962 |
| Q8BIP0 | 0.000303 | 0.000971 |
| O55106 | 0.000308 | 0.000985 |
| P68372 | 0.000313 | 0.000997 |
| P55284 | 0.000315 | 0.001001 |
| Q9DBL1 | 0.000325 | 0.001033 |
| O89053 | 0.000329 | 0.001041 |
| P70698 | 0.00033 | 0.001041 |
| Q9WU84 | 0.000335 | 0.001057 |
| Q9D1L0 | 0.000344 | 0.001082 |
| Q9CY58 | 0.000355 | 0.001115 |
| Q8CI94 | 0.000358 | 0.00112 |
| Q71FD7 | 0.000362 | 0.00113 |
| O09167 | 0.000362 | 0.00113 |
| P97290 | 0.000375 | 0.001166 |
| Q8BFP9 | 0.000385 | 0.001195 |
| Q9DCG9 | 0.000389 | 0.001207 |
| Q8BVQ5 | 0.000393 | 0.001214 |
| Q9Z2M7 | 0.000393 | 0.001214 |
| Q91VK1 | 0.000397 | 0.001223 |
| Q99K70 | 0.000398 | 0.001223 |
| P05132 | 0.0004 | 0.001228 |
| Q8VE95 | 0.000402 | 0.00123 |
| P51859 | 0.000409 | 0.001248 |
| Q9Z1J3 | 0.00041 | 0.001248 |
| P61358 | 0.00041 | 0.001248 |
| Q9R0H0 | 0.000415 | 0.00126 |
| Q9JKR6 | 0.00042 | 0.001273 |
| Q9D2G2 | 0.000433 | 0.00131 |
| Q99KP6 | 0.00044 | 0.001326 |
| Q61699 | 0.000458 | 0.00138 |
| Q61703 | 0.000465 | 0.001397 |
| O89023 | 0.000466 | 0.001399 |
| Q91ZJ5 | 0.000471 | 0.001409 |
| Q8QZS1 | 0.000478 | 0.001428 |
| P62313 | 0.000485 | 0.001445 |
| Q8CFI0 | 0.000486 | 0.001445 |
| P47811 | 0.000489 | 0.001452 |
| Q6IRU5 | 0.000503 | 0.00149 |
| Q78PY7 | 0.000504 | 0.00149 |
| P63325 | 0.000507 | 0.001495 |
| Q8BPN8 | 0.000513 | 0.001509 |
| P36552 | 0.000519 | 0.001526 |
| Q8R081 | 0.000521 | 0.001527 |
| Q61081 | 0.000522 | 0.001528 |
| Q9CQT1 | 0.00053 | 0.001546 |
| Q9CQ75 | 0.000545 | 0.001586 |
| P70670 | 0.000545 | 0.001586 |
| Q9Z0S1 | 0.000562 | 0.001632 |
| Q9CXW2 | 0.000574 | 0.001662 |
| P62077 | 0.000596 | 0.001723 |
| Q6PDM2 | 0.0006 | 0.00173 |
| Q61233 | 0.000604 | 0.00174 |
| Q9D1P0 | 0.000606 | 0.001743 |
| Q80X95 | 0.000609 | 0.001746 |
| Q00623 | 0.00061 | 0.001746 |
| Q8K3A0 | 0.000613 | 0.001751 |
| Q8BP47 | 0.000616 | 0.001756 |
| P18242 | 0.000617 | 0.001756 |
| Q99KK7 | 0.000627 | 0.001782 |
| Q9CQN7 | 0.00064 | 0.001815 |
| Q9R1Z8 | 0.000643 | 0.001818 |
| Q9CZY3 | 0.000644 | 0.001818 |
| Q922Q5 | 0.000652 | 0.001837 |
| Q8C0C7 | 0.000666 | 0.001873 |
| G5E861 | 0.000669 | 0.001877 |
| P03888 | 0.00067 | 0.001877 |
| Q07076 | 0.000671 | 0.001877 |
| Q91YY4 | 0.000673 | 0.00188 |
| Q8C176 | 0.000677 | 0.001886 |
| Q6X6Z7 | 0.000693 | 0.001927 |
| O35658 | 0.000702 | 0.001948 |
| Q8R146 | 0.000714 | 0.001978 |
| Q6PEB6 | 0.000717 | 0.001982 |
| P63280 | 0.000719 | 0.001982 |
| Q8K3W0 | 0.000721 | 0.001982 |
| P47962 | 0.000721 | 0.001982 |
| Q64737 | 0.000725 | 0.001989 |
| Q5M8N4 | 0.000731 | 0.002004 |
| Q9CR09 | 0.000733 | 0.002005 |
| Q9Z2W0 | 0.000746 | 0.002037 |
| Q99MR9 | 0.00075 | 0.002044 |
| Q8BFW7 | 0.000758 | 0.002062 |
| O88531 | 0.000765 | 0.002073 |
| P84244 | 0.000765 | 0.002073 |
| P97461 | 0.000802 | 0.002169 |
| Q9R269 | 0.000812 | 0.002193 |
| P15388 | 0.000826 | 0.002225 |
| Q9D4H8 | 0.000832 | 0.002239 |
| Q62000 | 0.000846 | 0.002269 |
| Q9JHL1 | 0.000847 | 0.002269 |
| Q00897 | 0.000853 | 0.002282 |
| P00405 | 0.000859 | 0.002293 |
| P19536 | 0.000876 | 0.002331 |
| Q921I1 | 0.000876 | 0.002331 |
| Q7TQD2 | 0.00088 | 0.002338 |
| P59999 | 0.000884 | 0.002343 |
| O09161 | 0.000892 | 0.002357 |
| Q8R1G2 | 0.000892 | 0.002357 |
| Q6ZQB6 | 0.000895 | 0.002359 |
| Q9D0R2 | 0.000908 | 0.002389 |
| P09528 | 0.000918 | 0.002412 |
| Q922B2 | 0.000937 | 0.002453 |
| Q9JHU2 | 0.000938 | 0.002453 |
| Q60932 | 0.000939 | 0.002453 |
| Q8BMS1 | 0.000941 | 0.002454 |
| Q9Z1N5 | 0.000948 | 0.002468 |
| P51885 | 0.000963 | 0.002502 |
| Q62348 | 0.000967 | 0.002508 |
| P14869 | 0.000969 | 0.002509 |
| P70398 | 0.000973 | 0.002511 |
| Q8CFX1 | 0.000973 | 0.002511 |
| Q8R2Y2 | 0.000977 | 0.002516 |
| P22892 | 0.000978 | 0.002516 |
| Q8CGB6 | 0.000984 | 0.002525 |
| Q9CQA3 | 0.000989 | 0.002535 |
| Q8K183 | 0.001021 | 0.002612 |
| P35486 | 0.00103 | 0.00263 |
| P56959 | 0.001059 | 0.0027 |
| Q922B1 | 0.001088 | 0.002769 |
| P63323 | 0.001093 | 0.002778 |
| Q7TNG8 | 0.001115 | 0.002829 |
| Q8K2B3 | 0.001123 | 0.002844 |
| Q99KI0 | 0.001128 | 0.002848 |
| E9QA28 | 0.001129 | 0.002848 |
| P63038 | 0.001171 | 0.00295 |
| Q811I0 | 0.001238 | 0.003114 |
| O35737 | 0.001246 | 0.003128 |
| Q61207 | 0.001249 | 0.00313 |
| O08756 | 0.001257 | 0.003143 |
| Q9QUH0 | 0.001268 | 0.003165 |
| Q9DBB5 | 0.001278 | 0.003186 |
| Q9DBR7 | 0.001318 | 0.00328 |
| Q9CQM5 | 0.001332 | 0.003311 |
| Q91VR5 | 0.00138 | 0.003422 |
| Q91VM9 | 0.001423 | 0.003524 |
| O70433 | 0.001437 | 0.003551 |
| P0C605 | 0.001453 | 0.003585 |
| O08585 | 0.001471 | 0.003625 |
| Q3V384 | 0.001482 | 0.003644 |
| Q6PAM1 | 0.001504 | 0.003693 |
| Q5XJY5 | 0.001553 | 0.003808 |
| Q9EPL8 | 0.001572 | 0.003848 |
| Q9JJW5 | 0.001595 | 0.003897 |
| P10639 | 0.001612 | 0.003932 |
| A6X935 | 0.001622 | 0.003949 |
| Q80SW1 | 0.001627 | 0.003949 |
| Q9D0M5 | 0.001627 | 0.003949 |
| Q6PB66 | 0.001673 | 0.004054 |
| Q9D855 | 0.001685 | 0.004075 |
| P62806 | 0.001709 | 0.004128 |
| Q9WTS4 | 0.001734 | 0.004181 |
| Q05920 | 0.001743 | 0.004195 |
| Q5SS00 | 0.001753 | 0.004213 |
| A2A791 | 0.001776 | 0.004262 |
| Q3ULJ0 | 0.001838 | 0.004403 |
| Q8CGY6 | 0.00185 | 0.004425 |
| O35226 | 0.001867 | 0.004457 |
| P04186 | 0.001919 | 0.004575 |
| P53994 | 0.00196 | 0.004665 |
| Q8C166 | 0.001996 | 0.004742 |
| Q8BVW0 | 0.002022 | 0.004796 |
| Q8K1R3 | 0.002083 | 0.004934 |
| P62996 | 0.002134 | 0.005045 |
| P57759 | 0.00219 | 0.005169 |
| P17742 | 0.002196 | 0.005176 |
| Q9CQV8 | 0.002264 | 0.005321 |
| B1AY13 | 0.002265 | 0.005321 |
| O35295 | 0.002274 | 0.005334 |
| Q91WM2 | 0.002287 | 0.005353 |
| O88696 | 0.00229 | 0.005353 |
| P10922 | 0.002304 | 0.005378 |
| Q8QZT1 | 0.002363 | 0.005507 |
| Q9CR62 | 0.00237 | 0.005514 |
| Q99LP6 | 0.002531 | 0.005879 |
| O55060 | 0.002557 | 0.005931 |
| Q8BKZ9 | 0.002623 | 0.00607 |
| Q8C5H8 | 0.002626 | 0.00607 |
| Q8JZU0 | 0.002629 | 0.00607 |
| Q8K1Z0 | 0.002672 | 0.006159 |
| O70400 | 0.002685 | 0.006179 |
| Q9DBL7 | 0.002742 | 0.0063 |
| P49935 | 0.002769 | 0.006353 |
| P48999 | 0.002783 | 0.006375 |
| Q9Z0X1 | 0.002865 | 0.006551 |
| P34884 | 0.002873 | 0.006559 |
| P62743 | 0.002953 | 0.006733 |
| Q64010 | 0.002976 | 0.00677 |
| P23116 | 0.002979 | 0.00677 |
| P80318 | 0.003012 | 0.006835 |
| Q9R0P9 | 0.003071 | 0.006957 |
| Q924M7 | 0.003075 | 0.006957 |
| P62827 | 0.003094 | 0.00699 |
| Q8K0E8 | 0.003107 | 0.007006 |
| P52825 | 0.003221 | 0.007253 |
| Q9JLJ2 | 0.003252 | 0.007312 |
| Q9D051 | 0.003313 | 0.007438 |
| P80313 | 0.003464 | 0.007765 |
| P32020 | 0.003484 | 0.007797 |
| Q9D0E1 | 0.00349 | 0.0078 |
| Q62417 | 0.003508 | 0.007827 |
| Q6PEV3 | 0.003528 | 0.007855 |
| Q61029 | 0.003531 | 0.007855 |
| Q99J39 | 0.003588 | 0.00797 |
| Q61133 | 0.003628 | 0.008046 |
| Q61598 | 0.003637 | 0.008055 |
| O54950 | 0.003667 | 0.008108 |
| Q9CR21 | 0.003679 | 0.008124 |
| Q3KQP7 | 0.003767 | 0.008304 |
| P42208 | 0.003779 | 0.008319 |
| Q06138 | 0.003903 | 0.008578 |
| O55142 | 0.004001 | 0.00877 |
| Q99L47 | 0.004002 | 0.00877 |
| O08759 | 0.00401 | 0.008774 |
| Q8CIB5 | 0.004174 | 0.009118 |
| P17751 | 0.004258 | 0.009289 |
| Q9QUG2 | 0.004309 | 0.009372 |
| Q52KB6 | 0.004311 | 0.009372 |
| O35660 | 0.004315 | 0.009372 |
| Q8K0D5 | 0.004381 | 0.009501 |
| Q99L13 | 0.004408 | 0.009544 |
| Q499X9 | 0.004504 | 0.00974 |
| O35386 | 0.004512 | 0.009742 |
| Q9D7J9 | 0.004584 | 0.009883 |
| P38060 | 0.004683 | 0.010082 |
| P48678 | 0.004812 | 0.010343 |
| Q99JI4 | 0.004866 | 0.010444 |
| Q91Y97 | 0.004881 | 0.010461 |
| Q8VIJ8 | 0.004952 | 0.010598 |
| Q9DC71 | 0.005029 | 0.010747 |
| Q8CIE6 | 0.005195 | 0.011085 |
| P48758 | 0.005396 | 0.011498 |
| Q8R086 | 0.005438 | 0.011571 |
| Q9DCL9 | 0.005446 | 0.011571 |
| Q7TSQ8 | 0.005569 | 0.011815 |
| P12382 | 0.005587 | 0.011821 |
| Q9JJ26 | 0.005588 | 0.011821 |
| Q9DC50 | 0.005597 | 0.011823 |
| Q80YV4 | 0.005625 | 0.011865 |
| Q9D0K2 | 0.005657 | 0.011915 |
| Q924T2 | 0.005671 | 0.011917 |
| O89020 | 0.005674 | 0.011917 |
| P62852 | 0.005758 | 0.012077 |
| Q99KR7 | 0.005863 | 0.012278 |
| P68254 | 0.005894 | 0.012326 |
| P26231 | 0.005921 | 0.012364 |
| Q9DBP5 | 0.00607 | 0.012658 |
| Q8K3Z0 | 0.006169 | 0.012845 |
| Q91VD9 | 0.006225 | 0.012944 |
| Q8BVI4 | 0.006276 | 0.013031 |
| P16546 | 0.006292 | 0.013048 |
| Q91V12 | 0.006421 | 0.013294 |
| Q91VN0 | 0.006446 | 0.013329 |
| Q8CEI1 | 0.006523 | 0.013452 |
| Q64475 | 0.006524 | 0.013452 |
| Q8K1H1 | 0.006568 | 0.013524 |
| Q9DCT8 | 0.006735 | 0.013848 |
| P34022 | 0.0068 | 0.013961 |
| Q8C050 | 0.006835 | 0.014014 |
| Q8K157 | 0.006885 | 0.014078 |
| Q9Z1D1 | 0.006897 | 0.014078 |
| Q99JY0 | 0.006904 | 0.014078 |
| P61027 | 0.006905 | 0.014078 |
| Q99PT1 | 0.006934 | 0.014118 |
| Q9D1N9 | 0.007032 | 0.014297 |
| Q3UIZ8 | 0.00713 | 0.014476 |
| Q61316 | 0.007231 | 0.014661 |
| Q8R1B4 | 0.007269 | 0.014718 |
| Q8VE38 | 0.007287 | 0.014735 |
| Q91WK5 | 0.007304 | 0.014749 |
| Q60823 | 0.007469 | 0.015061 |
| Q8CAF4 | 0.007693 | 0.015492 |
| Q8VEE1 | 0.007705 | 0.015495 |
| P08113 | 0.007807 | 0.015673 |
| Q8VCT4 | 0.007815 | 0.015673 |
| Q6PD19 | 0.00785 | 0.015721 |
| P80316 | 0.008095 | 0.016189 |
| Q03265 | 0.008112 | 0.016202 |
| Q91VA6 | 0.008366 | 0.016687 |
| Q62448 | 0.008401 | 0.016734 |
| O88685 | 0.008488 | 0.016883 |
| Q8BIW1 | 0.008667 | 0.017216 |
| Q11136 | 0.008696 | 0.017251 |
| Q5XPI3 | 0.008805 | 0.017443 |
| Q9DCN2 | 0.008824 | 0.017457 |
| P62242 | 0.008906 | 0.017596 |
| Q62422 | 0.008996 | 0.01775 |
| Q60854 | 0.009079 | 0.01789 |
| P97823 | 0.009222 | 0.018146 |
| P21981 | 0.009253 | 0.018184 |
| Q99JT2 | 0.009374 | 0.018397 |
| P35564 | 0.009498 | 0.018614 |
| O08553 | 0.009578 | 0.018747 |
| P62631 | 0.009672 | 0.018906 |
| P62071 | 0.009758 | 0.019048 |
| P14211 | 0.009778 | 0.019062 |
| P60229 | 0.009886 | 0.019247 |
| P38647 | 0.009974 | 0.019393 |
| P46935 | 0.010042 | 0.019499 |
| Q9WTP6 | 0.010143 | 0.019669 |
| P56394 | 0.010236 | 0.019824 |
| Q921G7 | 0.010397 | 0.020109 |
| Q9WUR2 | 0.010412 | 0.020111 |
| P51125 | 0.010455 | 0.020166 |
| Q9R1P0 | 0.010556 | 0.020335 |
| Q9DCV4 | 0.010966 | 0.021097 |
| P01837 | 0.011117 | 0.021355 |
| P48962 | 0.011129 | 0.021355 |
| Q8VCA8 | 0.011174 | 0.021408 |
| Q91WD5 | 0.011186 | 0.021408 |
| P21550 | 0.011287 | 0.021573 |
| P07724 | 0.011391 | 0.021744 |
| O35143 | 0.01141 | 0.021752 |
| Q60674 | 0.011585 | 0.022037 |
| P56480 | 0.01159 | 0.022037 |
| P31786 | 0.011953 | 0.022671 |
| Q9DCZ1 | 0.011954 | 0.022671 |
| P14152 | 0.012011 | 0.022749 |
| Q8VEM8 | 0.012026 | 0.022749 |
| Q9CZ30 | 0.012178 | 0.023007 |
| P56565 | 0.012277 | 0.023163 |
| O88844 | 0.012324 | 0.023222 |
| Q9R1L5 | 0.012394 | 0.023325 |
| P15532 | 0.012731 | 0.023928 |
| P58774 | 0.012806 | 0.024038 |
| Q99LC3 | 0.012838 | 0.024067 |
| Q8BRV5 | 0.01293 | 0.024209 |
| Q9R0P5 | 0.013046 | 0.024367 |
| P24527 | 0.013048 | 0.024367 |
| P62892 | 0.013099 | 0.024413 |
| P24549 | 0.013116 | 0.024413 |
| Q8BG95 | 0.013122 | 0.024413 |
| Q8BWT1 | 0.0132 | 0.024525 |
| P56380 | 0.013265 | 0.024615 |
| Q9CWH5 | 0.013469 | 0.024963 |
| Q8K4F5 | 0.013551 | 0.025084 |
| P48787 | 0.013683 | 0.025295 |
| P56382 | 0.0139 | 0.025664 |
| P07356 | 0.014033 | 0.025877 |
| Q3URE1 | 0.014092 | 0.025954 |
| P70699 | 0.014154 | 0.026034 |
| P06745 | 0.014211 | 0.026107 |
| Q07417 | 0.014438 | 0.026491 |
| Q9CZX8 | 0.01467 | 0.026884 |
| P42932 | 0.01473 | 0.02696 |
| P83940 | 0.014825 | 0.0271 |
| P26041 | 0.014913 | 0.027218 |
| P26039 | 0.014944 | 0.027218 |
| P08003 | 0.014946 | 0.027218 |
| P09671 | 0.014997 | 0.027277 |
| Q9DCD0 | 0.015093 | 0.027418 |
| A3KFX0 | 0.015265 | 0.027696 |
| Q9JII5 | 0.015505 | 0.028097 |
| Q6ZWM4 | 0.015658 | 0.028323 |
| Q9CWJ9 | 0.015668 | 0.028323 |
| O08528 | 0.015965 | 0.028823 |
| Q80W21 | 0.016044 | 0.028931 |
| P08228 | 0.016083 | 0.028965 |
| Q9R0Q6 | 0.016188 | 0.029119 |
| O35639 | 0.016214 | 0.029129 |
| Q9JLM9 | 0.016582 | 0.02974 |
| Q924X2 | 0.016594 | 0.02974 |
| Q60605 | 0.01662 | 0.02975 |
| Q3UGR5 | 0.01687 | 0.030123 |
| P63158 | 0.016888 | 0.030123 |
| P99024 | 0.01689 | 0.030123 |
| Q3ULD5 | 0.017199 | 0.030636 |
| P58771 | 0.017441 | 0.03103 |
| O09131 | 0.018021 | 0.032023 |
| Q8K2M0 | 0.018086 | 0.032099 |
| D3Z4I3 | 0.018242 | 0.032337 |
| P47915 | 0.018568 | 0.032875 |
| P48725 | 0.018611 | 0.032912 |
| Q8CHT0 | 0.018661 | 0.032934 |
| P01898 | 0.018669 | 0.032934 |
| Q9WUB3 | 0.018822 | 0.033164 |
| P08249 | 0.018954 | 0.033357 |
| Q91VI7 | 0.019028 | 0.033448 |
| Q9Z0R4 | 0.019453 | 0.034153 |
| P68368 | 0.019584 | 0.034342 |
| Q60936 | 0.019712 | 0.034526 |
| Q9JI91 | 0.019736 | 0.034527 |
| Q62261 | 0.019822 | 0.034635 |
| Q61292 | 0.019856 | 0.034654 |
| Q6P8J7 | 0.020186 | 0.035188 |
| P62821 | 0.020276 | 0.035302 |
| P24369 | 0.020316 | 0.03533 |
| Q8BQ33 | 0.020659 | 0.035884 |
| Q8VDQ1 | 0.020972 | 0.036371 |
| O55126 | 0.020989 | 0.036371 |
| Q9D0M3 | 0.021039 | 0.036414 |
| Q9D273 | 0.02115 | 0.036563 |
| Q9WTL7 | 0.021519 | 0.037157 |
| Q9Z2X8 | 0.022038 | 0.038009 |
| Q61239 | 0.02236 | 0.038519 |
| Q62426 | 0.022404 | 0.03855 |
| P17563 | 0.022821 | 0.039222 |
| Q8VCH8 | 0.023213 | 0.039848 |
| Q8R164 | 0.023298 | 0.039947 |
| P62900 | 0.023547 | 0.040326 |
| Q9D071 | 0.023592 | 0.040357 |
| P23506 | 0.024762 | 0.042309 |
| Q6A000 | 0.024812 | 0.042344 |
| Q99MN9 | 0.024842 | 0.042344 |
| P62737 | 0.024869 | 0.042344 |
| O35943 | 0.025122 | 0.042725 |
| Q8BSY0 | 0.025182 | 0.042777 |
| Q2PZL6 | 0.025361 | 0.043031 |
| Q9QZ23 | 0.025446 | 0.043125 |
| P28474 | 0.02556 | 0.043269 |
| Q8R0F8 | 0.025686 | 0.043432 |
| Q9WUM4 | 0.026007 | 0.043924 |
| Q689Z5 | 0.026122 | 0.044068 |
| Q8CI51 | 0.026238 | 0.044212 |
| O70468 | 0.02674 | 0.045007 |
| Q9JKF1 | 0.026818 | 0.045086 |
| O88653 | 0.027379 | 0.045959 |
| P48036 | 0.0274 | 0.045959 |
| Q70FJ1 | 0.027568 | 0.046189 |
| Q9DB27 | 0.027859 | 0.046622 |
| O09174 | 0.029133 | 0.048699 |
| Q3U5Q7 | 0.029346 | 0.048987 |
| A2ASS6 | 0.029372 | 0.048987 |
| Q9CWG8 | 0.029615 | 0.049335 |
| Q91WK1 | 0.029653 | 0.049342 |
| P62196 | 0.030078 | 0.049955 |
| Q99M87 | 0.030122 | 0.049955 |
| Q8VDJ3 | 0.030123 | 0.049955 |

| **MetaboliteID** | **p.value** | **FDR** |
| --- | --- | --- |
| PGF2a ethanolamide | 7,58E-07 | 0,000352 |
| trihomomethionine | 1,19E-05 | 0,002313 |
| 13(S)-HOTrE | 1,66E-05 | 0,002313 |
| (±)9-HpODE | 1,99E-05 | 0,002313 |
| Anserine | 3,70E-05 | 0,003284 |
| cis-Aconitic acid | 4,25E-05 | 0,003284 |
| Gamma-Glutamylcysteine | 8,07E-05 | 0,005352 |
| FA 16:0;O | 0,000106 | 0,005467 |
| [FA(18:0)-OH] | 0,000129 | 0,005468 |
| Acetyltaurine | 0,000133 | 0,005468 |
| FA 16:2 | 0,000162 | 0,005468 |
| Linolenic acid | 0,000162 | 0,005468 |
| (3-hydroxybutyrylcarnitine | 0,000166 | 0,005468 |
| 4-Trimethylammoniobutanal | 0,000177 | 0,005468 |
| 1D-myo-Inositol3-phosphate | 0,000191 | 0,005549 |
| LPE 16:0 | 0,000255 | 0,00697 |
| Uridine | 0,000308 | 0,007949 |
| C36-phenolphthiodiolenone A | 0,000343 | 0,008221 |
| Glucosamine 6-phosphate | 0,000354 | 0,008221 |
| 3-Dehydroxycarnitine | 0,0004 | 0,008833 |
| Ribose1_5-diphosphate | 0,000502 | 0,010589 |
| 3_5-Dihydroxy-phenylglycine | 0,000622 | 0,012543 |
| Maltose | 0,000796 | 0,015384 |
| LINALYL BUTYRATE | 0,000955 | 0,016766 |
| Cystathionine ketimine | 0,000969 | 0,016766 |
| Linoleic Acid | 0,000976 | 0,016766 |
| S-Lactoylglutathione | 0,001079 | 0,017784 |
| [FA22:5] | 0,001112 | 0,017784 |
| Pantetheine | 0,001198 | 0,018535 |
| Carnosine(beta-alanyl-L-histidine) | 0,001251 | 0,018722 |
| 4-(L-Alanin-3-yl)-2-hydroxy-cis_cis-muconate6-semialdehyde | 0,001374 | 0,018965 |
| Apigenin 7-sulfate | 0,001395 | 0,018965 |
| S-(3-oxo-3-carboxy-n-propyl)cysteine | 0,001406 | 0,018965 |
| Ile-cys | 0,001455 | 0,018965 |
| beta-Aspartylaspartic acid | 0,001478 | 0,018965 |
| Indole-2-carboxylic acid | 0,001577 | 0,018965 |
| N,N-Dihydroxy-5-(methylsulfanyl)norvaline | 0,001592 | 0,018965 |
| Succinic anhydride | 0,001594 | 0,018965 |
| 3-Anisic acid | 0,001745 | 0,020246 |
| CAR 18:1;O | 0,001869 | 0,021146 |
| PE 38:6 | 0,001934 | 0,021367 |
| LPE 22:6 | 0,002024 | 0,021842 |
| FA 14:1 | 0,002102 | 0,022163 |
| Octanoylcarnitine | 0,002291 | 0,023627 |
| Carnosine | 0,00243 | 0,024418 |
| Hydroxymethylphosphonate | 0,002492 | 0,024418 |
| 19-Hydroxytestosterone | 0,002534 | 0,024418 |
| Carnitine | 0,002583 | 0,024418 |
| 8(S),15(S)-DiHETE | 0,002631 | 0,024418 |
| Hexanoylcarnitine | 0,002764 | 0,025146 |
| Guanidineaceticacid | 0,003169 | 0,028274 |
| 2-(alpha-D-Galactosyl)-sn-glycerol3-phosphate | 0,003603 | 0,03103 |
| D-Alanine methyl ester | 0,003611 | 0,03103 |
| Adrenic acid | 0,003752 | 0,031562 |
| Glucose 1-phosphate | 0,003809 | 0,031562 |
| Oleic acid | 0,003892 | 0,031605 |
| 2-(2'-methylthio)ethylmalic-acid | 0,003951 | 0,031605 |
| 3-(3_4-Dihydroxypyridin-1-yl)-L-alanine | 0,004052 | 0,031863 |
| N-Hydroxy-2-acetamidofluorene | 0,004262 | 0,032211 |
| CAR 8:1 | 0,004267 | 0,032211 |
| Indole-3-acetaldoxime | 0,004318 | 0,032211 |
| Glutathione | 0,004464 | 0,032211 |
| Cholesterol sulfate | 0,004492 | 0,032211 |
| 3-(3_4-Dihydroxyphenyl)pyruvate | 0,004512 | 0,032211 |
| Lactose | 0,004645 | 0,032516 |
| Glycerylphosphorylethanolamine | 0,004695 | 0,032516 |
| Carbocysteine | 0,005428 | 0,036823 |
| LPC 22:6 | 0,005524 | 0,036823 |
| LPE 18:2 | 0,005555 | 0,036823 |
| Prostaglandin E2 | 0,00643 | 0,04202 |
| 3-Hydroxydecanoylcarnitine | 0,006813 | 0,043905 |
| PG 16:1 | 0,007387 | 0,046954 |
| 2,3-Bis(octanoyloxy)propanimidic acid | 0,007571 | 0,047444 |
| streptamine 5-phosphate | 0,007669 | 0,047444 |
| LPE 18:3 | 0,007839 | 0,047856 |
| N-Acetylglucosaminitol | 0,008172 | 0,049243 |
| Pseudouridine | 0,008436 | 0,049922 |
| 1-(Propyldisulfanyl)-1-(propylsulfinyl)propane | 0,008536 | 0,049922 |
| Diethyl phthalate | 0,008607 | 0,049922 |

| **Lipid_ID** | **p.value** | **FDR** |
| --- | --- | --- |
| PG 18:1_18:2_B | 1.02E-12 | 5.57E-10 |
| PC 18:0_22:4_A | 3.73E-11 | 8.09E-09 |
| PG 18:2_20:4 | 4.45E-11 | 8.09E-09 |
| PC 17:0_20:4_A | 8.45E-11 | 1.15E-08 |
| PC 20:4_22:6_A | 1.76E-10 | 1.92E-08 |
| PE 18:0_22:5_A | 6.41E-10 | 5.83E-08 |
| TG 18:1_18:2_20:0 | 4.71E-09 | 3.67E-07 |
| PG 18:2/18:2_B | 6.24E-09 | 3.99E-07 |
| BMP 18:2_18:2 | 6.57E-09 | 3.99E-07 |
| DG 18:1/18:1 | 8.87E-09 | 4.84E-07 |
| DG 18:1_22:5 | 1.00E-08 | 4.98E-07 |
| Cer 18:1;2O/26:0 | 1.57E-08 | 7.14E-07 |
| PC 13:0_22:4 | 2.34E-08 | 9.84E-07 |
| TG O-20:2_14:0_18:2 | 2.67E-08 | 9.85E-07 |
| PC 16:2_22:6 | 2.71E-08 | 9.85E-07 |
| TG 10:0_14:0_16:0 | 7.11E-08 | 2.43E-06 |
| Cer 18:1;2O/24:0 | 7.78E-08 | 2.50E-06 |
| PE 19:2_19:2 | 9.12E-08 | 2.77E-06 |
| PG 18:2_22:6_A | 1.23E-07 | 3.53E-06 |
| CE 18:3 | 1.51E-07 | 4.11E-06 |
| SM 24:0;2O/18:1 | 1.79E-07 | 4.67E-06 |
| LPE 20:4_B | 2.79E-07 | 6.92E-06 |
| CE 18:2 | 3.67E-07 | 8.72E-06 |
| HBMP 16:0_22:1_12:0 | 4.16E-07 | 9.48E-06 |
| DG 18:1_18:2 | 5.32E-07 | 1.12E-05 |
| PC O-34:2_A | 5.41E-07 | 1.12E-05 |
| LPC 22:4 | 5.54E-07 | 1.12E-05 |
| PC O-32:0_B | 5.78E-07 | 1.13E-05 |
| HexCer 9:0;2O/44:11 | 6.50E-07 | 1.19E-05 |
| PI 18:0_18:2 | 6.53E-07 | 1.19E-05 |
| PE P-16:0_22:4_A | 1.15E-06 | 2.01E-05 |
| PC 16:0_18:1 | 1.18E-06 | 2.01E-05 |
| PE P-18:1_18:1 | 1.24E-06 | 2.04E-05 |
| DG 16:0_18:1 | 1.52E-06 | 2.44E-05 |
| Cer 18:1;2O/26:1 | 1.59E-06 | 2.49E-05 |
| LPE 16:0 | 1.82E-06 | 2.77E-05 |
| SM 18:1;2O/24:1_B | 1.92E-06 | 2.84E-05 |
| PC O-36:4_A | 2.19E-06 | 3.09E-05 |
| PC 20:4_22:6_B | 2.21E-06 | 3.09E-05 |
| PE 16:0_18:0 | 2.27E-06 | 3.09E-05 |
| DG 16:0_22:5 | 2.57E-06 | 3.42E-05 |
| PE P-18:0_22:4_A | 3.54E-06 | 4.43E-05 |
| LPE 20:4_A | 3.58E-06 | 4.43E-05 |
| DG 16:0_18:2 | 3.65E-06 | 4.43E-05 |
| HBMP 16:0_18:1_18:2 | 3.73E-06 | 4.43E-05 |
| LPE 18:0 | 3.73E-06 | 4.43E-05 |
| PE 38:5 | 3.84E-06 | 4.47E-05 |
| PC 16:0_18:2_A | 4.05E-06 | 4.51E-05 |
| PC O-36:2 | 4.05E-06 | 4.51E-05 |
| PE P-20:0_20:4_A | 4.21E-06 | 4.59E-05 |
| PC O-34:1 | 5.11E-06 | 5.47E-05 |
| PE 18:3_22:6_A | 5.22E-06 | 5.48E-05 |
| SM 44:2;2O | 5.88E-06 | 6.06E-05 |
| PC 15:0_22:5 | 6.89E-06 | 6.97E-05 |
| SPB 25:0;2O | 7.57E-06 | 7.52E-05 |
| DG 18:2/18:2 | 8.20E-06 | 8.00E-05 |
| SM 18:1;2O/24:1_A | 8.99E-06 | 8.61E-05 |
| DG 18:2_20:1 | 9.77E-06 | 9.20E-05 |
| PC 44:5 | 1.19E-05 | 0.00011 |
| PI 36:4 | 1.33E-05 | 0.000121 |
| LPC 20:4 | 1.40E-05 | 0.000126 |
| SM 42:1;3O_B | 1.54E-05 | 0.000133 |
| PE P-18:1_22:6_A | 1.55E-05 | 0.000133 |
| PE 18:0/18:0 | 1.56E-05 | 0.000133 |
| PC O-38:0 | 1.72E-05 | 0.000145 |
| TG 16:0_16:1_17:0 | 1.83E-05 | 0.000152 |
| PC 34:4_A | 2.03E-05 | 0.000163 |
| SPB 24:0;2O | 2.04E-05 | 0.000163 |
| DG 15:0_24:0 | 2.06E-05 | 0.000163 |
| SM 34:2;2O | 2.12E-05 | 0.000165 |
| SM 42:3;2O | 2.17E-05 | 0.000165 |
| TG 16:0_16:0_16:1 | 2.17E-05 | 0.000165 |
| SM 22:0;2O/21:2 | 2.31E-05 | 0.000173 |
| HBMP 18:2_18:2_16:0_A | 2.43E-05 | 0.000179 |
| HBMP 18:2_18:2_16:0_B | 2.48E-05 | 0.00018 |
| PC 18:0_22:5_A | 2.61E-05 | 0.000188 |
| PC 18:0_22:5_B | 2.65E-05 | 0.000188 |
| PC 34:4_B | 2.69E-05 | 0.000188 |
| HexCer 14:2;2O/40:9 | 2.81E-05 | 0.000194 |
| TG 8:0_12:0_18:0 | 2.89E-05 | 0.000197 |
| LPE P-18:1_A | 3.01E-05 | 0.000203 |
| DG 16:1_18:2 | 3.18E-05 | 0.000211 |
| PE 36:5 | 3.28E-05 | 0.000216 |
| PI 18:2/18:2 | 3.41E-05 | 0.000221 |
| PC 18:3_22:6 | 3.45E-05 | 0.000221 |
| Cer 18:1;2O/23:0 | 3.48E-05 | 0.000221 |
| BMP 22:6_22:6 | 3.53E-05 | 0.000221 |
| PC 42:6_A | 3.64E-05 | 0.000226 |
| Cer 18:2;2O/22:1 | 3.75E-05 | 0.000229 |
| PC O-38:5 | 3.80E-05 | 0.000229 |
| PC 17:0_20:4_B | 3.84E-05 | 0.000229 |
| PE 34:0 | 3.85E-05 | 0.000229 |
| TG 15:0_16:1_24:0 | 4.10E-05 | 0.000241 |
| PC 36:0 | 4.16E-05 | 0.000242 |
| DG 16:0/16:0 | 4.24E-05 | 0.000242 |
| TG 18:1_18:2_18:2 | 4.25E-05 | 0.000242 |
| TG 16:0_16:0_18:2 | 4.30E-05 | 0.000242 |
| PC O-40:4 | 4.64E-05 | 0.000259 |
| PC 42:3 | 5.13E-05 | 0.000283 |
| PE P-18:0_20:4_A | 5.23E-05 | 0.000284 |
| LPC P-18:1 | 5.28E-05 | 0.000284 |
| TG 16:0_17:0_18:1 | 5.33E-05 | 0.000284 |
| PI 18:2_20:4 | 5.36E-05 | 0.000284 |
| TG 16:0_17:1_18:1 | 5.40E-05 | 0.000284 |
| PC 17:0_18:2 | 5.57E-05 | 0.000289 |
| PC 15:0_20:4 | 5.75E-05 | 0.000296 |
| PC 19:0_20:4_A | 6.27E-05 | 0.00032 |
| PC 14:0_17:0 | 6.56E-05 | 0.00033 |
| PE 17:0_22:6 | 6.64E-05 | 0.00033 |
| PE P-18:0_22:4_B | 6.65E-05 | 0.00033 |
| CE 18:1 | 8.12E-05 | 0.000398 |
| SM 18:1;2O/24:0 | 8.16E-05 | 0.000398 |
| DG 18:1_20:3 | 8.48E-05 | 0.00041 |
| TG 12:0_14:0_16:1 | 9.11E-05 | 0.000436 |
| SM 13:1;2O/21:1 | 9.21E-05 | 0.000437 |
| PC 14:0_22:6_A | 9.48E-05 | 0.000446 |
| Hex2Cer 39:1;2O | 0.000105 | 0.000487 |
| PC 18:0/18:0 | 0.000105 | 0.000487 |
| TG 18:1_18:2_20:1 | 0.000113 | 0.000517 |
| PC 22:5_22:5 | 0.000114 | 0.000519 |
| PC 15:1_24:6_A | 0.000116 | 0.000522 |
| PE 18:0_22:6_A | 0.000117 | 0.000522 |
| Cer 18:1;2O/22:0 | 0.000122 | 0.000541 |
| SM 18:1;2O/22:0 | 0.000123 | 0.000541 |
| TG 15:0_16:0_16:1 | 0.000133 | 0.000578 |
| PC 42:4 | 0.000133 | 0.000578 |
| TG 15:0_16:1_18:1 | 0.000136 | 0.000584 |
| LPE 18:1_B | 0.000147 | 0.000626 |
| PE P-16:0_22:6 | 0.000148 | 0.000628 |
| LPC 18:0 | 0.000155 | 0.000653 |
| TG 13:0_14:0_16:1 | 0.000159 | 0.000663 |
| TG 13:0_15:0_16:1 | 0.000166 | 0.000687 |
| PC 16:1_22:6 | 0.000169 | 0.000694 |
| PE 22:6_22:6 | 0.000183 | 0.000744 |
| PE 40:8 | 0.000184 | 0.000744 |
| PC 38:5 | 0.000186 | 0.000746 |
| PC 20:4_20:5 | 0.000188 | 0.000749 |
| SM 18:1;2O/20:0 | 0.000205 | 0.00081 |
| Cer 18:2;2O/24:1 | 0.000207 | 0.000812 |
| PC 34:3 | 0.00021 | 0.000821 |
| PC 17:0_18:1 | 0.000223 | 0.000859 |
| LPC 18:3 | 0.000223 | 0.000859 |
| TG 16:0_18:2_18:2 | 0.000227 | 0.000868 |
| LPI 18:0 | 0.000241 | 0.000914 |
| TG 18:2/18:2/18:2 | 0.000262 | 0.000985 |
| SM 20:1;2O/20:0 | 0.000268 | 0.000997 |
| SM 44:1;2O | 0.00027 | 0.000997 |
| DG 18:1_20:1 | 0.00027 | 0.000997 |
| PC 19:0_20:4_B | 0.000276 | 0.001002 |
| PC 34:0 | 0.000276 | 0.001002 |
| PC O-44:5 | 0.000277 | 0.001002 |
| PC 14:0_21:2 | 0.00028 | 0.001004 |
| PC 18:2_20:4 | 0.000286 | 0.001019 |
| PE 19:0_20:4 | 0.000301 | 0.001067 |
| TG O-16:0_16:0_18:2 | 0.000315 | 0.001109 |
| SM 32:1;2O/11:0 | 0.000317 | 0.001109 |
| PC 22:6_22:6_A | 0.000319 | 0.001111 |
| PC O-40:9 | 0.000323 | 0.001118 |
| Cer 18:1;2O/24:1 | 0.000352 | 0.00121 |
| PG 16:0_18:1_A | 0.00036 | 0.001228 |
| PC O-40:8_A | 0.000363 | 0.00123 |
| HexCer 18:1;2O/23:0 | 0.000371 | 0.00125 |
| PC O-38:4 | 0.000387 | 0.001298 |
| LPE P-18:1_B | 0.000392 | 0.001306 |
| PC 16:1_18:2 | 0.000398 | 0.001319 |
| SM 18:1;2O/23:0 | 0.000401 | 0.00132 |
| TG 15:1_16:1_16:1 | 0.000408 | 0.001336 |
| Hex2Cer 18:1;2O/22:0 | 0.000442 | 0.001436 |
| LPC O-16:1 | 0.000451 | 0.001458 |
| PE 18:2_22:6_A | 0.000455 | 0.001461 |
| TG 18:1_18:2_22:1 | 0.000469 | 0.001496 |
| PC 15:0_18:1 | 0.000495 | 0.001571 |
| TG 16:0_16:1_16:1 | 0.00051 | 0.00161 |
| TG 12:0_15:1_16:1 | 0.000523 | 0.001642 |
| PC 19:0_18:1_B | 0.000546 | 0.001699 |
| PC 38:6 | 0.000548 | 0.001699 |
| SM 44:3;2O | 0.000566 | 0.001746 |
| Cer 18:1;2O/21:0 | 0.000574 | 0.00176 |
| LPE 18:1_A | 0.000608 | 0.001855 |
| PI 38:5 | 0.000614 | 0.001862 |
| PC 38:2 | 0.000632 | 0.001906 |
| PI 18:1_18:2 | 0.000655 | 0.001965 |
| SM 24:1;2O/17:0 | 0.000671 | 0.002003 |
| PE 18:3_22:6_B | 0.000689 | 0.002043 |
| PI 16:0_18:2 | 0.000712 | 0.002101 |
| PE 34:3 | 0.000716 | 0.002101 |
| PC 18:2_22:6_B | 0.000745 | 0.002176 |
| PE 16:0_18:1 | 0.000756 | 0.002196 |
| TG 14:1_16:1_16:1 | 0.000777 | 0.002244 |
| PC 22:5_22:6_A | 0.000805 | 0.002314 |
| PC 16:0_18:2_B | 0.000811 | 0.002318 |
| PE P-20:0_20:4_B | 0.000828 | 0.002354 |
| MLCL 18:1_18:1_18:2 | 0.000887 | 0.002511 |
| LPE P-16:0 | 0.000893 | 0.002514 |
| PI 40:6_A | 0.000902 | 0.002525 |
| PC 19:0_18:2_B | 0.000956 | 0.002662 |
| PE P-18:0_20:4_B | 0.000972 | 0.002693 |
| PE 21:1_18:5 | 0.000993 | 0.002728 |
| LPC 24:1 | 0.000994 | 0.002728 |
| PC O-30:0 | 0.001007 | 0.002749 |
| DG 18:0_18:1 | 0.001022 | 0.002776 |
| PC 20:5_20:5 | 0.001057 | 0.002856 |
| TG 14:0_14:1_16:1 | 0.00107 | 0.002877 |
| TG 14:0_15:0_16:1 | 0.001077 | 0.002882 |
| TG 13:0_16:1_16:1 | 0.001089 | 0.002899 |
| Cer 18:1;2O/19:0 | 0.001103 | 0.002924 |
| PC 34:5 | 0.001143 | 0.003016 |
| Cer 18:2;2O/18:0 | 0.001177 | 0.003089 |
| PC 15:1_24:5 | 0.001201 | 0.003126 |
| TG 14:1_16:0_16:1 | 0.001202 | 0.003126 |
| LPC 24:0 | 0.001281 | 0.003315 |
| PI 18:0_20:3 | 0.001369 | 0.003526 |
| CE 20:3 | 0.001388 | 0.003558 |
| PC 18:0_22:6_A | 0.001446 | 0.00368 |
| DG O-18:1_18:2 | 0.001449 | 0.00368 |
| TG 14:0_16:0_16:1 | 0.001488 | 0.003761 |
| PI 18:1_20:4 | 0.001526 | 0.00384 |
| PC 22:1_24:6 | 0.001556 | 0.003897 |
| CE 16:1 | 0.001598 | 0.003984 |
| PE 16:0_22:6 | 0.001643 | 0.004078 |
| CE 16:0 | 0.001728 | 0.004265 |
| PC 16:0_16:1 | 0.001734 | 0.004265 |
| SM 42:0;3O | 0.001837 | 0.004498 |
| TG 18:1_18:2_22:6 | 0.001851 | 0.004513 |
| TG 18:1_18:2_24:1 | 0.001953 | 0.00474 |
| PG 18:2_22:6_C | 0.002017 | 0.004874 |
| PC 14:0_21:1 | 0.002093 | 0.005034 |
| SPB 26:0;2O | 0.002109 | 0.005051 |
| PE P-20:1_22:6 | 0.002121 | 0.005056 |
| LPC P-16:0 | 0.002217 | 0.005263 |
| TG 14:1_16:1_18:1 | 0.002232 | 0.005277 |
| PC O-36:4_B | 0.002267 | 0.005336 |
| LPE 18:2_A | 0.002303 | 0.005397 |
| PC 18:1_22:6_A | 0.00232 | 0.005414 |
| PI 17:0_20:4 | 0.00237 | 0.005506 |
| PI 16:0_20:4 | 0.002396 | 0.005543 |
| PE 40:5 | 0.002493 | 0.005744 |
| PE 18:2_22:6_B | 0.00254 | 0.005826 |
| LPC 16:1_B | 0.002567 | 0.005865 |
| SM 42:1;3O_A | 0.002719 | 0.006187 |
| PC 19:2_18:4 | 0.002735 | 0.006196 |
| LPC 17:0 | 0.002777 | 0.006257 |
| PC 15:1_26:6_B | 0.002785 | 0.006257 |
| TG 15:0_16:1_16:1 | 0.002831 | 0.006336 |
| LPC 20:2 | 0.002865 | 0.006384 |
| PC 17:0_22:5 | 0.002914 | 0.006467 |
| PC 44:4 | 0.003079 | 0.006807 |
| PE 18:1_20:4 | 0.003107 | 0.00684 |
| PE 18:2_22:5 | 0.003243 | 0.00711 |
| SM 18:2;2O/23:0 | 0.003255 | 0.00711 |
| PC 18:0_18:1_A | 0.003292 | 0.007162 |
| PE 18:1_22:6_B | 0.003336 | 0.007227 |
| PC O-42:5 | 0.003377 | 0.007289 |
| LPC 16:1_A | 0.003456 | 0.007428 |
| PC 40:0 | 0.003477 | 0.007445 |
| SM 12:1;2O/27:0 | 0.003522 | 0.007495 |
| PC 16:0_17:0 | 0.003528 | 0.007495 |
| PI 40:7 | 0.003555 | 0.007523 |
| PC 16:0_22:6 | 0.003682 | 0.007733 |
| PE P-17:0_22:6 | 0.003687 | 0.007733 |
| Cer 18:2;2O/23:0 | 0.003697 | 0.007733 |
| DG 18:0_22:6 | 0.00373 | 0.007773 |
| PC O-34:3_B | 0.003765 | 0.007816 |
| PE P-16:0_18:2 | 0.003884 | 0.008004 |
| PC 20:3_22:6 | 0.003885 | 0.008004 |
| PE 18:0_20:3 | 0.003929 | 0.008055 |
| SM 40:2;2O | 0.003939 | 0.008055 |
| PC 15:0_22:6 | 0.003989 | 0.008127 |
| SM 30:3;2O/12:0 | 0.004155 | 0.008433 |
| TG 16:0_18:1_18:2 | 0.004214 | 0.008522 |
| SM 20:1;2O/21:1 | 0.004319 | 0.008699 |
| Cer 18:1;2O/20:0 | 0.004334 | 0.008699 |
| CL 18:2/18:2/18:2/18:2 | 0.004372 | 0.008712 |
| PI 40:6_B | 0.004376 | 0.008712 |
| DG 22:1_20:3 | 0.004388 | 0.008712 |
| Cer 18:2;2O/24:2 | 0.004469 | 0.008836 |
| PE 18:0_22:6_B | 0.004483 | 0.008836 |
| PC O-38:6_B | 0.004743 | 0.009316 |
| PS 16:0_22:6 | 0.004766 | 0.009328 |
| TG 18:1_22:5_22:6 | 0.004815 | 0.00939 |
| TG 18:2_18:2_22:6 | 0.004842 | 0.009408 |
| SM 21:1;2O/16:0 | 0.004972 | 0.009626 |
| PC 16:0/16:0 | 0.00508 | 0.009802 |
| PC 22:5_22:6_B | 0.005191 | 0.009979 |
| HexCer 15:3;2O/36:8 | 0.005288 | 0.01013 |
| PC O-38:6_A | 0.005451 | 0.010406 |
| LPC 14:0 | 0.005489 | 0.010442 |
| PC 16:0_20:4 | 0.005589 | 0.010595 |
| PE 18:1_18:2_A | 0.005769 | 0.010898 |
| TG 16:0_16:1_21:0 | 0.005954 | 0.011211 |
| PC 14:0_22:6_B | 0.005986 | 0.011219 |
| PC 32:1 | 0.006 | 0.011219 |
| DG 17:1_18:1 | 0.006154 | 0.011468 |
| PG 16:0_18:2_B | 0.006208 | 0.01153 |
| PC 15:1_24:6_B | 0.006267 | 0.011599 |
| PE P-16:0_18:1 | 0.00631 | 0.011639 |
| PE 16:1_22:6 | 0.006348 | 0.011639 |
| PC 18:0_22:6_B | 0.006352 | 0.011639 |
| PE 34:1 | 0.006534 | 0.011931 |
| LPC 18:2_A | 0.006661 | 0.012122 |
| LPC O-18:1 | 0.006907 | 0.012529 |
| PG 15:0_18:1 | 0.006942 | 0.012551 |
| LPE 18:2_B | 0.007009 | 0.01263 |
| Cer 18:1;2O/18:0 | 0.007493 | 0.013458 |
| PC 19:0_22:6_B | 0.007594 | 0.013594 |
| LPC 16:0 | 0.007627 | 0.013608 |
| DG 16:0_18:0 | 0.007696 | 0.013678 |
| PC 32:3 | 0.007716 | 0.013678 |
| DG 18:0_20:1 | 0.007808 | 0.013796 |
| PE 16:0_18:2 | 0.008169 | 0.014388 |
| PC 15:1_26:6_A | 0.008199 | 0.014394 |
| PG 18:0_18:2 | 0.008572 | 0.015 |
| PC 30:0 | 0.008677 | 0.015136 |
| PC O-42:6 | 0.008802 | 0.015304 |
| PC 16:0_18:0 | 0.009072 | 0.015681 |
| TG 16:0_18:2_18:3 | 0.009076 | 0.015681 |
| TG 18:2_18:2_20:1 | 0.009861 | 0.016985 |
| SM 21:1;2O/19:1 | 0.009922 | 0.017035 |
| PE P-18:0_18:1_B | 0.010469 | 0.017919 |
| PI 16:1_18:2 | 0.010874 | 0.018536 |
| LPE 22:6 | 0.010898 | 0.018536 |
| PE P-18:0_22:6_B | 0.010982 | 0.018621 |
| LPC 18:2_B | 0.011177 | 0.018893 |
| TG 18:2_22:5_22:6 | 0.011481 | 0.019332 |
| PE 15:0_22:6 | 0.011507 | 0.019332 |
| PE 34:2 | 0.011621 | 0.019464 |
| PC 14:0_23:0 | 0.011816 | 0.019729 |
| HexCer 40:1;2O | 0.011934 | 0.019866 |
| PE P-19:0_22:6 | 0.012079 | 0.020046 |
| PC 22:6_22:6_B | 0.012136 | 0.02008 |
| SM 18:1;2O/21:0 | 0.012358 | 0.020384 |
| MLCL 16:2_18:0_18:2 | 0.012656 | 0.020814 |
| PC 18:1_18:2 | 0.012698 | 0.02082 |
| PC 18:0_18:2 | 0.013296 | 0.021735 |
| LPC 18:1_A | 0.013374 | 0.021798 |
| TG O-18:1_18:1_18:1 | 0.013691 | 0.022249 |
| Hex2Cer 18:1;2O/24:0 | 0.013982 | 0.022654 |
| PE 18:2/18:2 | 0.014122 | 0.022812 |
| Cer 18:2;2O/20:0 | 0.014913 | 0.024019 |
| Cer 18:2;2O/22:0 | 0.015133 | 0.024302 |
| PE P-20:0_22:6_B | 0.015783 | 0.025271 |
| Cer 18:2;2O/21:0 | 0.015891 | 0.025356 |
| Cer 18:0;2O/22:0 | 0.015929 | 0.025356 |
| LPC 22:6_B | 0.016231 | 0.025762 |
| LPC 22:0 | 0.016303 | 0.025802 |
| MLCL 18:2_18:2_18:2 | 0.016498 | 0.026034 |
| PC 19:0_18:1_A | 0.016897 | 0.026588 |
| PC 16:2_18:2 | 0.01726 | 0.027081 |
| DG 16:0_17:0 | 0.017578 | 0.027454 |
| Cer 18:0;2O/18:0 | 0.017599 | 0.027454 |
| PE 18:1_18:2_B | 0.018213 | 0.028331 |
| PC 32:2 | 0.018874 | 0.029276 |
| PC 15:0_18:2 | 0.019123 | 0.029578 |
| GM3 40:1;2O | 0.019742 | 0.03045 |
| PC 38:0 | 0.02083 | 0.032037 |
| PC 20:1_22:6 | 0.020934 | 0.032107 |
| TG 18:2_18:2_18:3 | 0.021191 | 0.03241 |
| PC 38:1 | 0.021423 | 0.032674 |
| LPC 20:1_B | 0.022048 | 0.033532 |
| Cer 18:2;2O/19:0 | 0.022474 | 0.034048 |
| HexCer 13:0;2O/42:12 | 0.022511 | 0.034048 |
| LPC 18:1_B | 0.02377 | 0.035851 |
| PE P-18:0_18:1_A | 0.023867 | 0.035899 |
| PC 17:0_24:1 | 0.024402 | 0.036603 |
| PC 40:1 | 0.024559 | 0.03665 |
| PC 20:0_19:2 | 0.024568 | 0.03665 |
| PC 16:0_22:5 | 0.02495 | 0.037119 |
| PC 18:0_22:4_B | 0.026221 | 0.038903 |
| CE 18:0 | 0.026519 | 0.03924 |
| PE 29:0_13:1 | 0.026622 | 0.039285 |
| PC O-34:0 | 0.026882 | 0.039563 |
| SM 15:2;2O/24:0 | 0.026964 | 0.039577 |
| PC 18:0_22:6;O | 0.027202 | 0.039819 |
| Cer 18:1;2O/17:0 | 0.027322 | 0.039888 |
| PC 40:3 | 0.027474 | 0.040002 |
| PE P-18:0_20:5 | 0.027993 | 0.040649 |
| Hex2Cer 18:1;2O/20:0 | 0.028396 | 0.041029 |
| LPG 18:1 | 0.028457 | 0.041029 |
| PC O-34:3_A | 0.02848 | 0.041029 |
| CE 20:1 | 0.028839 | 0.041437 |
| CE 21:2 | 0.029194 | 0.041838 |
| PC 38:8 | 0.029301 | 0.04188 |
| PC 36:3 | 0.029499 | 0.042053 |
| PE 20:1_22:6 | 0.029584 | 0.042065 |
| PC 16:1_20:4 | 0.029878 | 0.042373 |
| PC 9:0_34:6 | 0.030257 | 0.042798 |
| PC 18:0_22:5_D | 0.0311 | 0.043877 |
| PE 18:0_18:2_A | 0.031251 | 0.043977 |
| PC 44:6 | 0.0316 | 0.044353 |
| Cer 18:0;2O/20:0 | 0.031851 | 0.044591 |
| PE 18:0_18:2_B | 0.03315 | 0.046292 |
| PC 19:0_22:6_A | 0.033803 | 0.047082 |
| PG 16:0_18:1_B | 0.035905 | 0.049884 |
